# Loss of Crb2b-lf leads to anterior segment defects in old zebrafish

**DOI:** 10.1242/bio.047555

**Published:** 2020-02-11

**Authors:** Satu Kujawski, Cátia Crespo, Marta Luz, Michaela Yuan, Sylke Winkler, Elisabeth Knust

**Affiliations:** Max Planck Institute of Molecular Cell Biology and Genetics, Pfotenhauerstrasse 108, 01307 Dresden, Germany

**Keywords:** Development, Polarity, Cornea, Lens, Iris

## Abstract

Defects in the retina or the anterior segment of the eye lead to compromised vision and affect millions of people. Understanding how these ocular structures develop and are maintained is therefore of paramount importance. The maintenance of proper vision depends, among other factors, on the function of genes controlling apico-basal polarity. In fact, mutations in polarity genes are linked to retinal degeneration in several species, including human. Here we describe a novel zebrafish *crb2b* allele (*crb2b^e40^*), which specifically affects the *crb2b* long isoform. *crb2b^e40^* mutants are viable and display normal ocular development. However, old *crb2b^e40^* mutant fish develop multiple defects in structures of the anterior segment, which includes the cornea, the iris and the lens. Phenotypes are characterised by smaller pupils due to expansion of the iris and tissues of the iridocorneal angle, an increased number of corneal stromal keratocytes, an abnormal corneal endothelium and an expanded lens capsule. These findings illustrate a novel role for *crb2b* in the maintenance of the anterior segment and hence add an important function to this polarity regulator, which may be conserved in other vertebrates including humans.

## Abstract (precis)

**Summary:** Loss of the apico-basal polarity regulator Crb2b-lf does not affect retinal development but leads to ocular anterior segment defects in old zebrafish.

## INTRODUCTION

The vertebrate eye is a complex organ, the development and function of which depend on an intricate interaction of multiple tissues to ensure vision (Graw, 2010; Heavner and Pevny, 2012). Structures of the ocular anterior segment (AS) – the cornea, lens and iris – focus incoming light onto the photoreceptor cells (PRCs) of the retina. The signal generated in PRCs by the activation of the phototransduction cascade is passed on to the other cell types of the neural retina – horizontal, bipolar, amacrine, ganglion cells and Müller glia – which process and transmit it to the brain compartment processing visual information (the visual cortex in higher vertebrates or the optic tectum in lower vertebrates). Defects in either the retina or the AS lead to compromised vision or even blindness. Thus, understanding how these structures form and are maintained is of utmost importance for unravelling the origin of human eye diseases.

The development and maintenance of the vertebrate retina has been extensively studied (Amini et al., 2017; Hoon et al., 2014; Stenkamp, 2015). In contrast, less is known on the molecular mechanisms that govern the formation and homeostasis of the AS (reviewed in Cvekl and Tamm, 2004; Gage and Zacharias, 2009; Gould et al., 2004; Idrees et al., 2006; Soules and Link, 2005; Sowden, 2007). In the mature eye, the cornea and the lens refract and transmit light on to the retina, whereas the pigmented iris controls the amount of light allowed to enter. The AS also regulates intraocular pressure by balancing aqueous humour production by the ciliary body with its outflow via specialised tissues at the iridocorneal angle (ICA), the region where the cornea and the iris join (Gould et al., 2004; Gray et al., 2009; Soules and Link, 2005). The biology of the AS is of high interest, as many common eye diseases originate in defective AS structures. These include glaucoma, lens cataracts and corneal dystrophies (Cvekl and Tamm, 2004; Gould et al., 2004; Idrees et al., 2006; Soules and Link, 2005).

The zebrafish has developed into an excellent organism for modelling human ocular diseases originating in the AS. First, the development of the various tissues of the AS and their adult morphology, and the mechanisms regulating aqueous humour outflow, have been described in detail. The zebrafish eye shows an overall conservation in retinal and AS anatomy compared with other vertebrate eyes, including the human eye (Akhtar et al., 2008; Gray et al., 2009; Puzzolo et al., 2018; Richardson et al., 2017; Soules and Link, 2005; Zhao et al., 2006). Second, the cornea, iris and lens originate from corresponding cell lineages in zebrafish and mammals (Greiling and Clark, 2009; Soules and Link, 2005). Third, the developing and mature cornea resembles that of humans both in structure and gene expression (Akhtar et al., 2008; Takamiya et al., 2015), and the mature lens is structurally and functionally similar to that of humans, albeit showing some differences in developmental mechanisms (Dahm et al., 2007; Greiling and Clark, 2009). Tissues of the fish ICA show conservation in anatomy and ultrastructure between zebrafish and mammals, though they exhibit distinct species-specific modifications (Chen et al., 2008; Gray et al., 2009). Fourth, several zebrafish mutants with defects in AS tissues have been identified and add to our knowledge of genes involved in AS morphogenesis (Aose et al., 2017; Fadool et al., 1997; Gath and Gross, 2019; Hendee et al., 2018; Ji et al., 2016; Krall et al., 2018; Lee et al., 2012; Murphy et al., 2011; Pathania et al., 2014; Schonthaler et al., 2010; Takamiya et al., 2015; Vihtelic and Hyde, 2002; Vihtelic et al., 2001). Finally, the zebrafish offers many experimental advantages. Due to rapid *ex utero* development in a transparent chorion, eye morphogenesis can be observed *in vivo*. Cell lineages can be tracked in great detail, using various transgenic and eye mutant lines (Amini et al., 2019; Greiling and Clark, 2009; Icha et al., 2016; Sidhaye and Norden, 2017; Weber et al., 2014). Screening for potential drugs is also feasible in large numbers using zebrafish embryos (Wiley et al., 2017; Zon and Peterson, 2005).

Besides retinal neurons, some AS structures, such as the cornea and the lens, differentiate from polarised epithelia (Melo et al., 2017; Soules and Link, 2005; Zhao et al., 2006). Epithelia are characterised by a pronounced apico-basal polarity, manifested by the asymmetric distribution of proteins, organelles and various specialised junctions that ensure tissue integrity (e.g. tight and adherens junctions). Apico-basal polarity is established and maintained by four evolutionarily conserved polarity protein complexes, the apical Par3-Par6-aPKC and the Crumbs complexes, and the basolateral Scribble-Lgl-Dlg and the Coracle-Yurt modules (Assémat et al., 2008; Bazellieres et al., 2009; Bulgakova and Knust, 2009; Elsum et al., 2012; Flores-Benitez and Knust, 2016; Goldstein and Macara, 2007; Laprise et al., 2009; Rodriguez-Boulan and Macara, 2014; Tepass, 2012; Yamanaka and Ohno, 2008). These protein complexes subdivide the cell into an apical and a basal compartment, separated by cellular junctions. Any change in the levels of polarity proteins, achieved through loss or overexpression, can lead to aberrant compartment formation, complete loss of polarity and/or missing or mispositioned junctions, which eventually results in compromised tissue integrity and a faulty epithelium (Macara et al., 2014; Rodriguez-Boulan and Macara, 2014; St Johnston and Sanson, 2011; Tellkamp et al., 2014).

Given the epithelial origin of several eye tissues, it is not surprising that mutations in polarity genes are causally related to human eye diseases. Among these are retinitis pigmentosa 12 (RP12) and Leber congenital amaurosis (LCA), severe ocular dystrophies leading to PRC degeneration caused by mutations in *CRUMBS1* (*CRB1*) or *CRB2* (Chen et al., 2018; den Hollander et al., 1999; reviewed in Bujakowska et al., 2012; Slavotinek, 2016). Similarly, mutations in mouse *Crb1/Crb2* (Alves et al., 2014, 2013; Mehalow et al., 2003; van de Pavert et al., 2004) and in *Drosophila crb* (Chartier et al., 2012; Johnson et al., 2002; Mishra et al., 2012; Spannl et al., 2017; recently reviewed in Pichaud, 2018), result in photoreceptor degeneration. Zebrafish *crb2a* (*oko meduzy*, *ome*) is required early in the retinal neuroepithelium to maintain apico-basal polarity and epithelial integrity. As a consequence, retinal lamination does not proceed normally in the absence of *crb2a* function (Malicki and Driever, 1999; Omori and Malicki, 2006). Furthermore, knockdown (KD) of *crb2b*, the second *crb2* zebrafish paralogue, by morpholinos (MOs) has been shown to result in smaller PRC inner segments (IS) (Omori and Malicki, 2006). In addition, mutations in polarity genes, including *crb2a*, induce defects in the integrity of the corneal epithelium and affect the layering of the cornea (Beyer et al., 2010). Together these data show that Crb proteins are paramount regulators of polarity, cell compartmentalisation and tissue integrity also in epithelia-derived tissues of the eye.

Here, we set out to study the role of zebrafish *crb2b* in eye development in more detail. *crb2b* encodes two protein isoforms, Crb2b-long form (Crb2b-lf) and Crb2b-short form (Crb2b-sf), which are the result of alternative transcription start sites (Zou et al., 2012). A *crb2b* allele with an early stop codon in the long isoform generated here does not affect the development of the retina nor that of the AS. Interestingly, however, old *crb2b-lf* mutant fish exhibit complex abnormalities in the cornea, iris and lens of the AS. These findings underscore the importance of zebrafish in eye research and in the future may contribute to our understanding of the origin of human diseases.

## RESULTS

### *crb2b-lf* mutants are homozygous viable and show normal ocular development

**Fig. 1.**
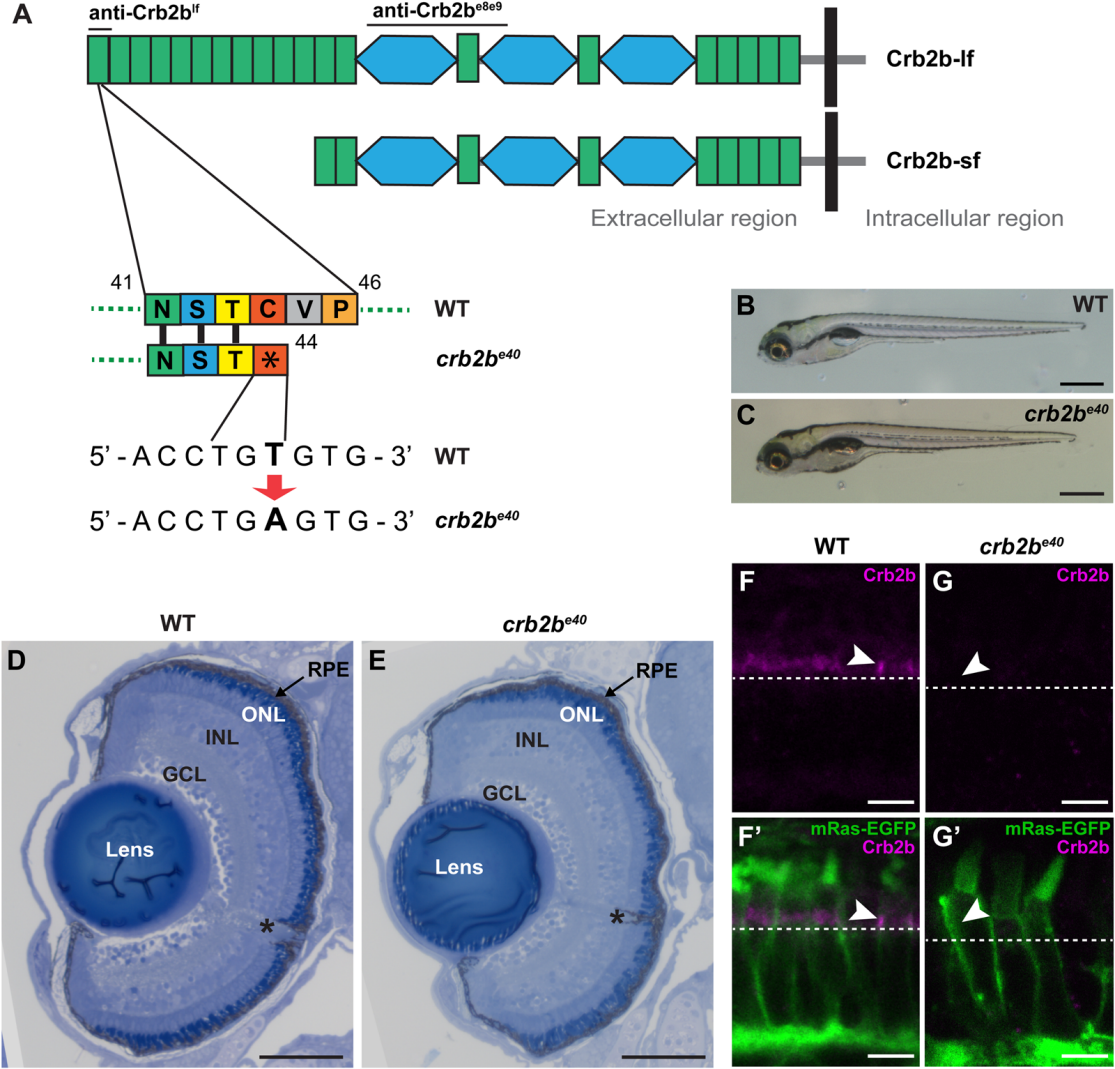
***crb2b^e40^* mutants are homozygous viable and develop normal eyes.** (A) Schematic illustration of the long and short isoforms of Crb2b, Crb2b-lf and Crb2b-sf. Green rectangles, EGF-like domains; light blue hexagons, domains with similarity to the globular domain of laminin A (LamG domain). Bars above the protein indicate the regions used as antigens for anti-Crb antibodies. In the *crb2b^e40^* allele, a T to A transversion translates to an early stop codon at amino acid position 44 of the protein, resulting in truncated Crb2b-lf. (B,C) Brightfield images of WT (B) and *crb2b^e40^* (C) zebrafish larvae at 5 dpf. Mutant larvae have overall normal appearance. (D,E) Transverse retinal sections stained with Toluidine Blue show normal lamination of the retina of *crb2b^e40^* (E) larvae in comparison to WT (D) at 5 dpf. Asterisk denotes the optic nerve. GCL, ganglion cell layer; INL, inner nuclear layer; ONL, outer nuclear layer; RPE, retinal pigment epithelium. (F–G′) Confocal images of transverse retinal sections of larvae at 3 dpf stained with rabbit anti-Crb2b^e8e9^ in the Tg(*bactin*:mRas-EGFP) background. Crb2b is detected in the IS of WT PRCs (F,F′, white arrowheads), but not in *crb2b^e40^* mutant PRCs (G,G′). White dashed lines mark the position of the outer limiting membrane. Scale bars: (B,C) 1 mm; (D,E) 100 µm; (F–G′) 5 µm.

So far, the function of zebrafish *crb2b* in the eye has been studied during early retinal development by *crb2b* MO-mediated knockdown (Omori and Malicki, 2006) or overexpression of dominant negative forms in the adult retina (Fu et al., 2018; Zou et al., 2012). To study the function of *crb2b* during ocular differentiation and maintenance in more detail, we screened for *crb2b* mutants using TILLING (Targeting Induced Local Lesions in Genomes) in a previously generated zebrafish ENU (N-ethyl-N-nitrosourea)-mutagenesis collection (Winkler et al., 2011). TILLING revealed a *crb2b* allele with a point mutation (T to A, nucleotide 349 on NM_001045162.1) in exon 2, which generates an early stop codon (C44X) and translates to a protein truncated within the first EGF-like domain of Crb2b (Fig. 1A). We named this allele *crb2b^e40^*. *crb2b* is known to encode two protein isoforms, Crb2b long form (Crb2b-lf) and Crb2b short form (Crb2b-sf) (Fig. 1A) (Zou et al., 2012). As the *crb2b-sf* transcriptional start site is located in the seventh intron of *crb2b* (Zou et al., 2012), the mutation in the *crb2b^e40^* allele is expected to be Crb2b-lf specific.

At 5 dpf, homozygous *crb2b^e40^* mutant larvae show no obvious external defects (Fig. 1B,C) and *crb2b^e40^* mutants survive into adulthood and breed normally, as has been reported previously for a very similar allele carrying a stop codon in the Crb2b-lf signal peptide (Hazime and Malicki, 2017). Furthermore, histological analyses of tissue organisation in *crb2b^e40^* eyes at 5 dpf did not show any apparent defect in the overall structure of the retina, the retinal pigment epithelium (RPE) or the lens (Fig. 1D,E). To confirm the absence of Crb2b protein in *crb2b^e40^* mutant eyes we generated a rabbit anti-Crb2b serum (anti-Crb2b^e8e9^) against two epitopes in the Crb2b ECD, encoded by exons 8 and 9. Hence, anti-Crb2b^e8e9^ recognises both the long and the short form of Crb2b (Fig. 1A). We stained wild-type (WT) and mutant retinas at 3 dpf with anti-Crb2b^e8e9^ antibodies in the Tg(*bactin*:mRas-EGFP) background (Cooper et al., 2005), which allows for the visualisation of PRC shape and compartmentalisation (Crespo and Knust, 2018). In WT PRCs at 3 dpf, a clear Crb2b^e8e9^ signal is detected in the subapical region (SAR) of the IS, directly apical to the outer limiting membrane (OLM) (Fig. 1F,F′). This signal is missing in the retina of mutants (Fig. 1G,G′).

The lack of an anti-Crb2b^e8e9^ antibody signal in the *crb2b^e40^* background suggests that Crb2b-lf is the main Crb2b isoform expressed during development, as anti-Crb2b^e8e9^ recognises both Crb2b isoforms. This conclusion is supported by results on *crb2b-lf* and *crb2b-sf* mRNA expression obtained at different stages of WT development (Fig. S1). In WT fish during early development (1–5 dpf), *crb2b-sf* transcript levels were low in whole fish extracts and could not be reliably quantified (data not shown). In later developmental stages *crb2b-lf* mRNA levels in the eye remain constant (Fig. S1A), but *crb2b-sf* transcript levels are upregulated as the fish mature from larva (14 dpf) to adult (3 m) (Fig. S1B; a lower delta C_t_ value indicates upregulation of mRNA). Importantly, *crb2b-sf* transcripts were not upregulated in *crb2b^e40^* mutant retinas (Fig. S1B). From these data we conclude that Crb2b-lf is the main Crb2b isoform produced during early development and is not necessary for normal morphogenesis of the eye, including retinal lamination.

### *crb2b^e40^* mutant PRCs are polarised and show proper maturation

**Fig. 2.**
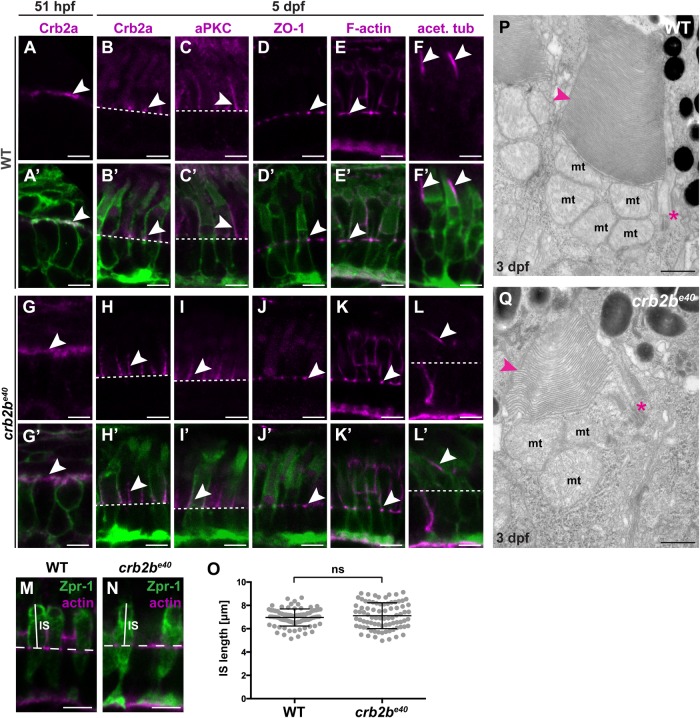
***crb2b^e40^* mutant PRCs have normal morphology and polarity.** (A–L′) Immunostaining of transverse sections of WT (A–F′) and *crb2b^e40^* (G–L′) retinas in the Tg(*bactin*:mRas-EGFP) background at 51 hpf (A,A′,G,G′) and 5 dpf (B–F′,H–L′). Crb2a localises on the entire apical membrane in WT (A,A′) and *crb2b^e40^* mutants (G,G′) PRC precursors of this stage (arrowheads). At 5 dpf, Crb2a and aPKC localise to the IS (arrowheads) in WT (B,B′,C,C′) and in mutant cells (H,H′,I,I′). ZO-1 and F-actin localise to the outer limiting membrane (OLM; white arrowheads), both in WT (D–E′) and mutants (J–K′). A well-formed axoneme (white arrowheads) is detected in retinal sections in both WT (F,F′) and *crb2b^e40^* (L,L′) fish by acetylated tubulin antibody staining. White dashed lines indicate OLM. (M,N) Transverse retinal sections of WT (M) and *crb2b^e40^* (N) larvae at 3 dpf stained with Zpr-1 antibody to mark the cell body of double-cone PRCs (green) and phalloidin to mark the OLM (magenta). The solid white lines indicate IS height, the dashed white lines mark the position of the OLM. (O) Quantification of IS length (µm) in both WT and *crb2b^e40^* PRCs. IS length was measured from the level of the OLM to the base of the OS (see M,N). At least 20 cells from three independent retinas were measured. Statistical significance was calculated by *t*-test (unpaired, with equal s.d., two-tailed). ns, not significant (*P=*0.3053). (P,Q) Transmission electron micrographs of WT (P) and *crb2b^e40^* (Q) OSs at 3 dpf. The overall ultrastructure of cilia (magenta asterisks) and OS membrane discs (magenta arrowheads) is preserved in mutant PRCs. mt, mitochondrium. Scale bars: (A–N) 5 µm; (P,Q) 1 µm.

Loss of polarity proteins has been associated with defects in the establishment and maintenance of PRC polarity and junctional complexes (Hsu and Jensen, 2010; Krock and Perkins, 2014; Omori and Malicki, 2006). Therefore, we evaluated the polarity of *crb2b^e40^* PRCs by analysing Crb2a and aPKC localisation in *crb2b^e40^*;Tg(*bactin*:mRas-EGFP) and WT Tg(*bactin*:mRas-EGFP) retinas. At 51 hpf, PRC precursors show a columnar shape characteristic for this stage of development, with Crb2a localised to the entire apical membrane in both mutant and in WT tissue (Fig. 2A,G, arrowhead) (Crespo and Knust, 2018). By 5 dpf, the overall shape of the mutant cells does not differ from WT as visualised by EGFP fluorescence expressed by the transgene. Crb2a (Fig. 2B,H, arrowhead) and aPKC (Fig. 2C,I, arrowhead) localisation is restricted to the IS in both WT and mutant PRCs. In addition, ZO-1 (Fig. 2D,J, arrowhead) as well as F-actin (phalloidin staining, Fig. 2E,K, arrowhead) correctly localised to the level of the OLM in WT and mutant tissue. As MO-mediated knockdown of Crb2b was reported to result in shorter ISs (Omori and Malicki, 2006), we measured IS length in *crb2b^e40^* mutant PRCs at 3 dpf (Fig. 2M–O). No significant difference in mutant versus WT PRCs was observed (Fig. 2O). These data suggest no major aberration in cell compartmentalisation in mutant cells. This conclusion was corroborated by transmission electron microscopy (TEM). In *crb2b^e40^* PRCs at 3 dpf, mitochondria localised to the ellipsoid region of the IS as in WT (Fig. 2P,Q), and junctions were properly positioned (data not shown).

Finally, we asked whether the most apical part of PRCs, the outer segment (OS), is properly formed in *crb2b^e40^* eyes. The OS is a modified cilium, and Crb proteins are known to play a role in ciliogenesis (Fan et al., 2007; Hazime and Malicki, 2017; Omori and Malicki, 2006). Acetylated tubulin staining (Fig. 2F,L, arrowheads) did not reveal any difference in the appearance of the axoneme of the connecting cilium between WT and mutant PRCs. Analysis of OS by TEM (Fig. 2P,Q) showed that in *crb2b^e40^* retinas, the basal body, the transition zone and the axoneme, with its nine doublets of microtubules, do not show any obvious defects at 3 dpf (Fig. 2Q, asterisk, and data not shown). OS membrane stacks appeared normal in *crb2b^e40^* mutants at this time point (Fig. 2Q, arrowhead). Furthermore, no obvious difference was detected in the size of EGFP-labelled OSs (Fig. S2) visualised by membrane-targeted EGFP in Tg(*bactin*:mRas-EGFP) PRCs. Altogether, these data show that Crb2b-lf is not necessary for PRC polarisation or maturation, as *crb2b^e40^* PRCs display normal localisation of apical polarity and junctional proteins as well as a normal compartmentalisation.

### Crb2b is expressed in the developing AS

Interestingly, junctional and apico-basal polarity factors, including Crb2a, have been reported to play a role also in the development of the cornea and the lens (Beyer et al., 2010). These two tissues, together with the ciliary body, the iris and the tissues of the ICA, comprise the ocular AS. Therefore, we asked whether Crb2b plays a role in the development or maintenance of AS structures by analysing its expression and the AS phenotype of *crb2b^e40^* mutant fish.

**Fig. 3.**
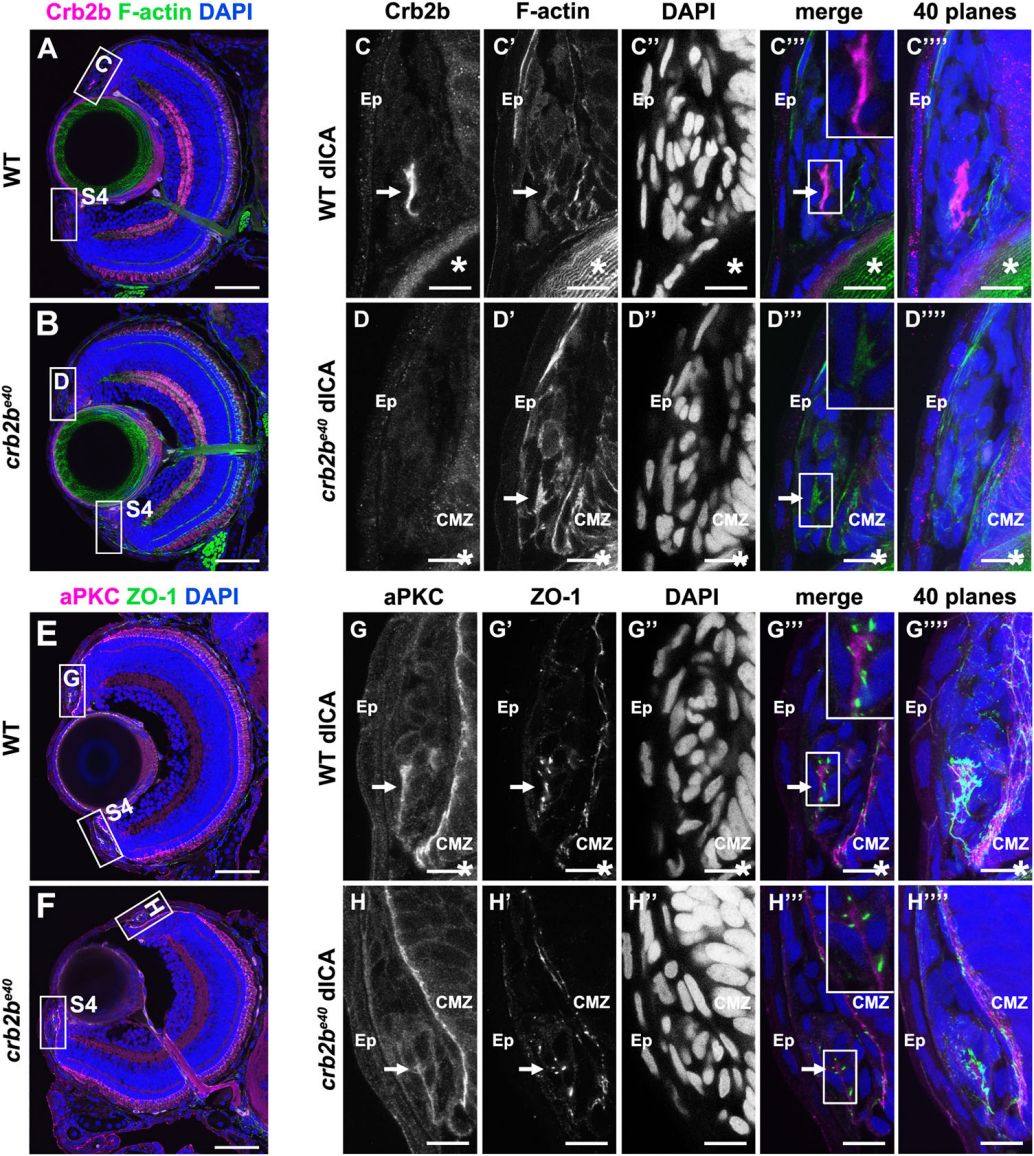
**Crb2b is expressed in polarised cells of the ICA at 5 dpf.** Immunostaining of transverse cryosections through the eye. (A,B,E,F) Overview of a WT (A,E) and a mutant (B,F) eye at 5 dpf. Higher magnifications of the boxed regions in the dorsal ICA are shown in C–D‴′ (WT) and G–H‴′ (*crb2b^e40^*). Higher magnifications of the boxed regions in the ventral ICA are shown in Fig. S4. Crb2b staining (anti-Crb2b^e8e9^) is detected in a cluster of cells in the WT ICA (C) but not in the mutant ICA (D). F-actin (C′,D′; visualised by phalloidin), aPKC (G,H) and ZO-1 (G′,H′) appear apically enriched (C′,D′). Arrows point to the cluster of polarised Crb2b expressing cells, and asterisks mark the lens. dICA, dorsal iridocorneal angle; Ep, epidermis; CMZ, ciliary marginal zone. Scale bars: (A,B,E,F) 50 µm; (C–D‴′, G–H‴′) 10 µm.

Since *crb2b* mRNA has been shown to be expressed in the AS at 7 dpf (Hsu et al., 2006), we analysed Crb2b protein expression in cryosections of larval ocular tissues (Fig. S3), using two different Crb2b antibodies, which detect either both isoforms (anti-Crb2b^e8e9^) or only the long isoform (anti-Crb2b^lf^) (see Fig. 1A). Either antibody stains a small population of Crb2b-expressing cells in tissues of the developing iridocorneal angle, on both the dorsal (Fig. 3A,C and Fig. S3) and ventral side (Fig. S4A). We did not detect any specific Crb2b signal in other tissues of the AS. Co-labelling with other markers revealed an asymmetric accumulation of filamentous actin (Fig. 3C′ and Fig. S4A′), aPKC (Fig. 3G and Fig. S4C) and ZO-1 (Fig. 3G′ and Fig. S4C′), pointing to an apico-basal polarity in these cells. In many cases the apical domains were facing each other, suggesting a rosette or sheet-like arrangement of the cells (Fig. 3C′″,G′″). In *crb2b^e40^* mutant larvae, no Crb2b was detected in the ICA by either anti-Crb2b^e8e9^ (Fig. 3B,D and Fig. S4B), anti-Crb2b^lf^ or an anti-Crb antibody that detects all Crumbs proteins (Omori and Malicki, 2006) (data not shown). Phalloidin (Fig. 3D′ and Fig. S4B′), aPKC (Fig. 3H and Fig. S4D) and ZO-1 (Fig. 3H′ and Fig. S4D′) staining clearly identified the presence of these cells in the mutant, but revealed no difference in localisation of these markers. These data show that Crb2b-lf is expressed in a small group of cells in the developing AS.

### Loss of Crb2b-lf results in eye defects in old fish

**Fig. 4.**
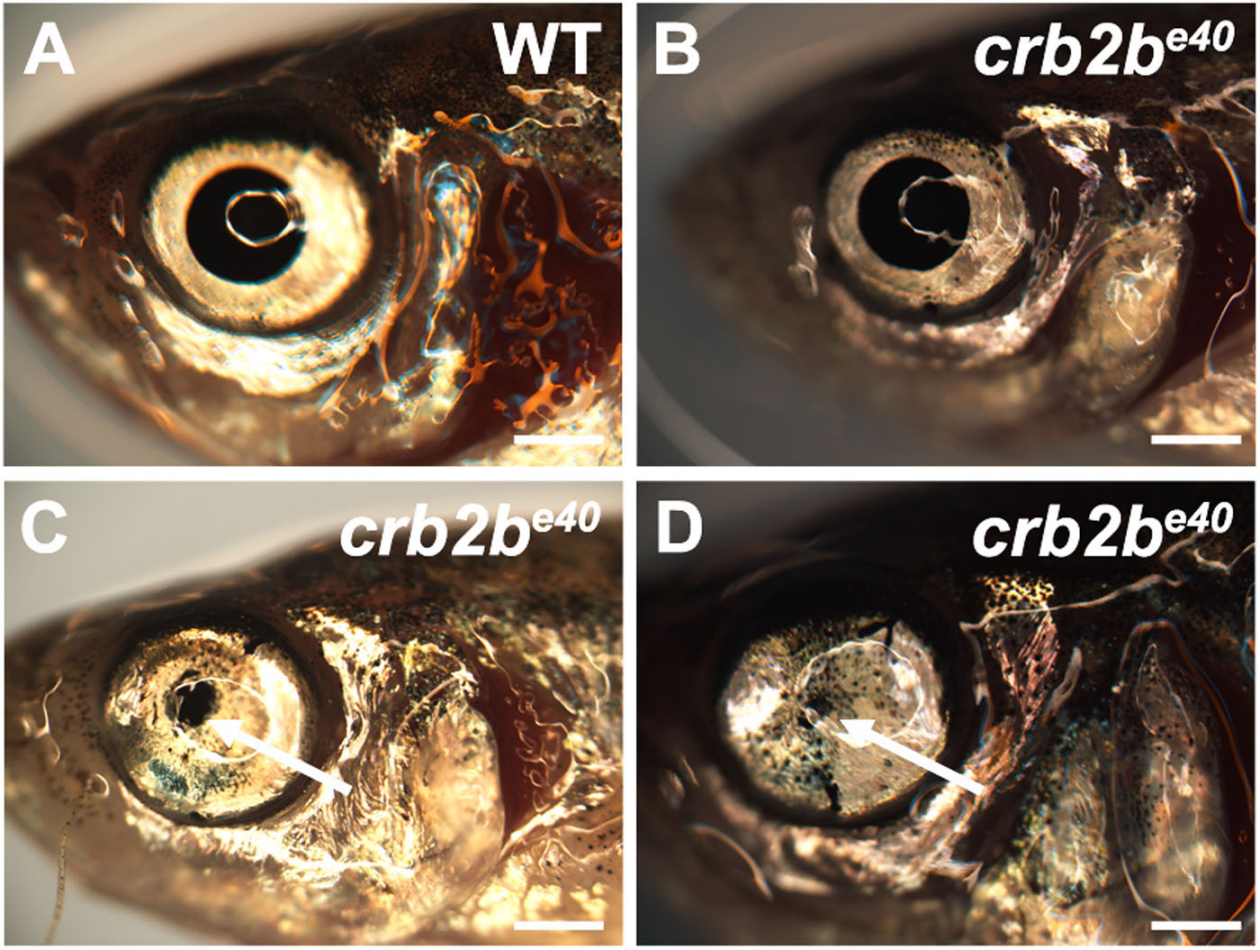
**Old *crb2b^e40^* mutants have abnormal AS tissues.** Brightfield images of old adult WT (A) and *crb2b^e40^* (B–D) eyes. Arrows in C and D point to a reduced pupil. Scale bars: 1 mm.

Loss of Crb2b-lf does not seem to influence the early development of the AS, as the structure of *crb2b^e40^* larval eyes is normal (Fig. 1E). However, old mutant fish displayed an eye phenotype with incomplete penetrance and expressivity, which sometimes was observed only unilaterally. In a total of 87 *crb2b^e40^* adults older than 2.5 years, 34% (30 fish) displayed external defects of varying severity in the AS of the eye (Fig. 4). The phenotype is characterised by a decrease in pupil size (Fig. 4B–D). In extreme cases, the lens (2 of 5 eyes dissected) or the complete eye (4 of 87 fish analysed) was lost. Such phenotype was normally not detected in WT adults.

### Loss of Crb2b-lf results in ICA tissue overgrowth in old fish

**Fig. 5.**
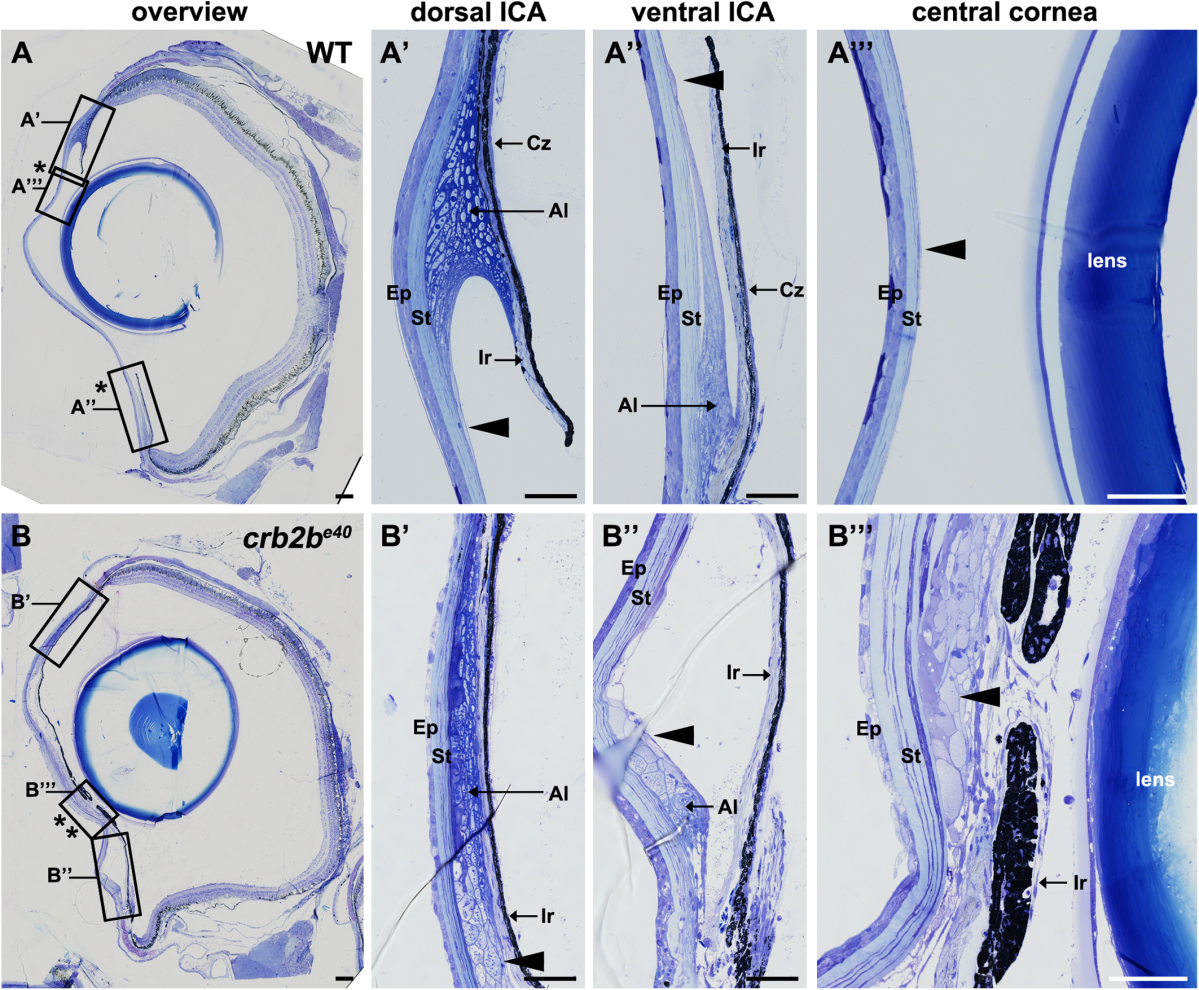
**Loss of Crb2b-lf leads to an overgrowth of the iris in old fish.** Toluidine Blue-stained transverse retinal sections of WT (A–A″′) and *crb2b^e40^* (B–B″′) adult zebrafish. (A–A″′) Overview of the whole WT eye (A) shows the normal appearance of the pupil (bordered by two asterisks). Higher magnification of WT dorsal (A′) and ventral (A″) iridocorneal angles shows the morphology of the iris, annular ligament cells and endothelial cells lining the ligament (arrowhead). A″′ shows the structure of the cornea, with the corneal endothelium cells being barely visible (A″′, arrowhead). (B–B″′) Overview of the whole eye of a mutant fish (B) demonstrates a decrease in pupil size (bordered by two asterisks) due to overgrowth of ICA tissue and an expansion of the iris towards the centre of the cornea (B″′). Multiple layers of cells line the posterior surface of the cornea (B″′, arrowhead). These cells appear to extend from the annular ligament (B′,B″ arrowheads). Ep, corneal epidermis; St, corneal stroma; Al, annular ligament; Ir, iris; Cz, ciliary zone. Scale bars: (A,B) 100 µm; (A′–A″′) and (B′–B″′) 50 µm.

**Fig. 6.**
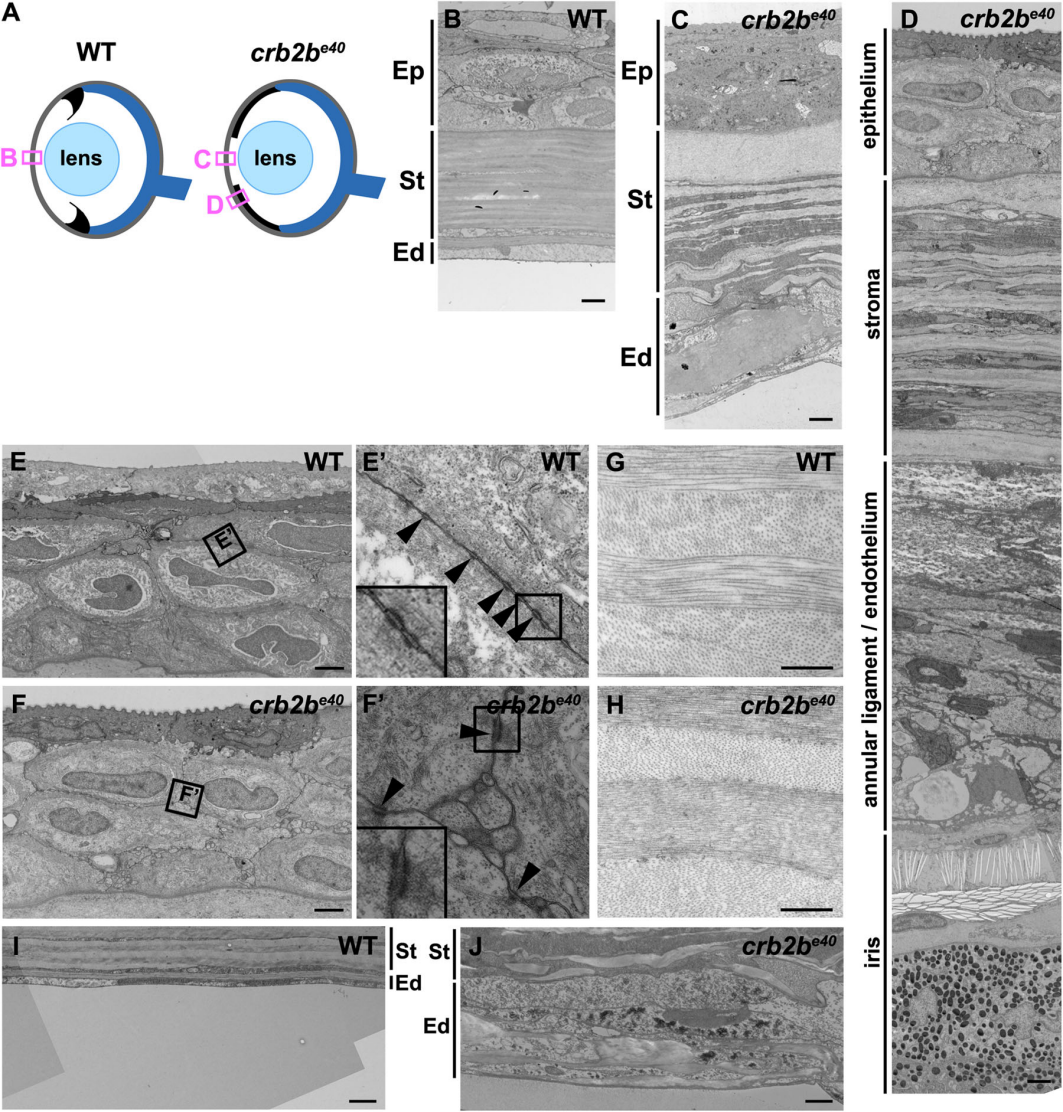
**Loss of Crb2b-lf leads to corneal abnormalities in old fish.** (A) Simplified schematic of a WT (left) and a *crb2b^e40^* (right) adult eye phenotype. Boxes denote regions of TEM images shown in B–D. (B–D) TEM transverse sections through the central cornea of adult WT (B) and *crb2b^e40^* fish (C,D) without (C) and with (D) iris expansion. (E–J) Higher magnification of the corneal layers in WT and mutant fish. Mutant corneal epidermal cells (F) do not appear different from their WT counterparts (E), and junctions (boxed regions in E,F) are visible in both (E′,F′, arrowheads). In the corneal stroma, collagen fibres are orthogonally layered both in the WT (G) and in the mutant (H). The endothelium is monolayered in WT (B,I), but appears as a multi-layered tissue in the mutant (C,J; labelled as annular ligament/endothelium in C), next to the elongated iris (D). Ep, corneal epidermis; St, corneal stroma; Ed, corneal endothelium. Scale bars: (B–F,I,J) 2 µm; (G,H) 0.5 µm.

To better understand the phenotype in old *crb2b^e40^* adult fish, we performed histological analyses of WT and mutant eye tissues. Toluidine Blue staining of transverse ocular sections revealed defects in multiple tissues of the AS, including the ICA, the cornea and the lens. The adult WT zebrafish ICA is formed by the iris, the ciliary zone and the annular ligament (Fig. 5A′,A″). The pigmented iris (Ir) borders the lens and leaves an opening that forms the pupil (Fig. 5A, asterisks demarcate the limits of the pupil). The base of the iris, called the ciliary zone (Cz), is formed by non-pigmented epithelial cells (Soules and Link, 2005). The annular ligament (Al), which runs circumferentially, is characterised by a fibrous meshwork due to deposition of glycoprotein aggregates (Fig. 5A′,A″). A thin monolayer of endothelial cells lines the anterior side of the iris and covers the surface of the annular ligament (Fig. 5A′,A″, arrowheads), extending into the endothelial layer of the cornea (Fig. 5A″′, arrowhead). In *crb2b^e40^* mutant fish, the iris expands towards the centre of the eye, resulting in a reduced size of the pupil (Fig. 5B, asterisks; see Fig. 4C,D). In addition, the annular ligament in mutant eyes is continu­ous with abnormal cell clusters on the inner side of the cornea, where a monolayered endothelium is found in WT (Fig. 5B′–B″′, arrowheads, Fig. 6D). These data show that the loss of Crb2b leads to an abnormal expansion of ICA tissues.

### Lack of Crb2b-lf results in corneal abnormalities in old fish

Besides the iris, the cornea was also strongly affected in *crb2b^e40^* adult fish. To further characterise this phenotype, we compared overall corneal tissue morphology of WT and mutant eyes in Toluidine Blue stained histological sections (Fig. 5A″′,B″′) and the corneal ultrastructure by TEM (Fig. 6). In the cornea of WT fish, a multi-layered corneal epithelium, formed by four to six layers of interdigitated cells connected by desmosomes (Soules and Link, 2005; Zhao et al., 2006), protects the corneal stromal layer (Fig. 6B, Ep; Fig. 6E,E′). The stroma is composed of orthogonally arranged layers of collagen fibres (Fig. 6B, St; Fig. 6G), which are laid down by flattened keratocytes residing within the stroma. A flat, monolayered, polarised corneal endothelium lines the stroma on the posterior side of the cornea (Fig. 6B, Ed; Fig. 6I) (Soules and Link, 2005).

In *crb2b^e40^* mutants, the corneal epithelium did not display major abnormalities in layering or in morphology (Figs 5B″′ and 6F), and cells were connected by desmosomal junctions as in WT (Fig. 6F′). While stromal collagen fibres were properly aligned in mutants similar to those in WT (Fig. 6H), the corneal endothelium appeared abnormal (Figs 5B″′ and 6C,D in comparison to A″′,B). Multiple layers of cells were present on the posterior surface of the mutant cornea (Fig. 6C,J), where a monolayer of endothelial cells is normally found (Fig. 6B,I). Based on their morphology and their lightly stained cytoplasm, these cells have characteristics of corneal endothelial cells (Soules and Link, 2005).

**Fig. 7.**
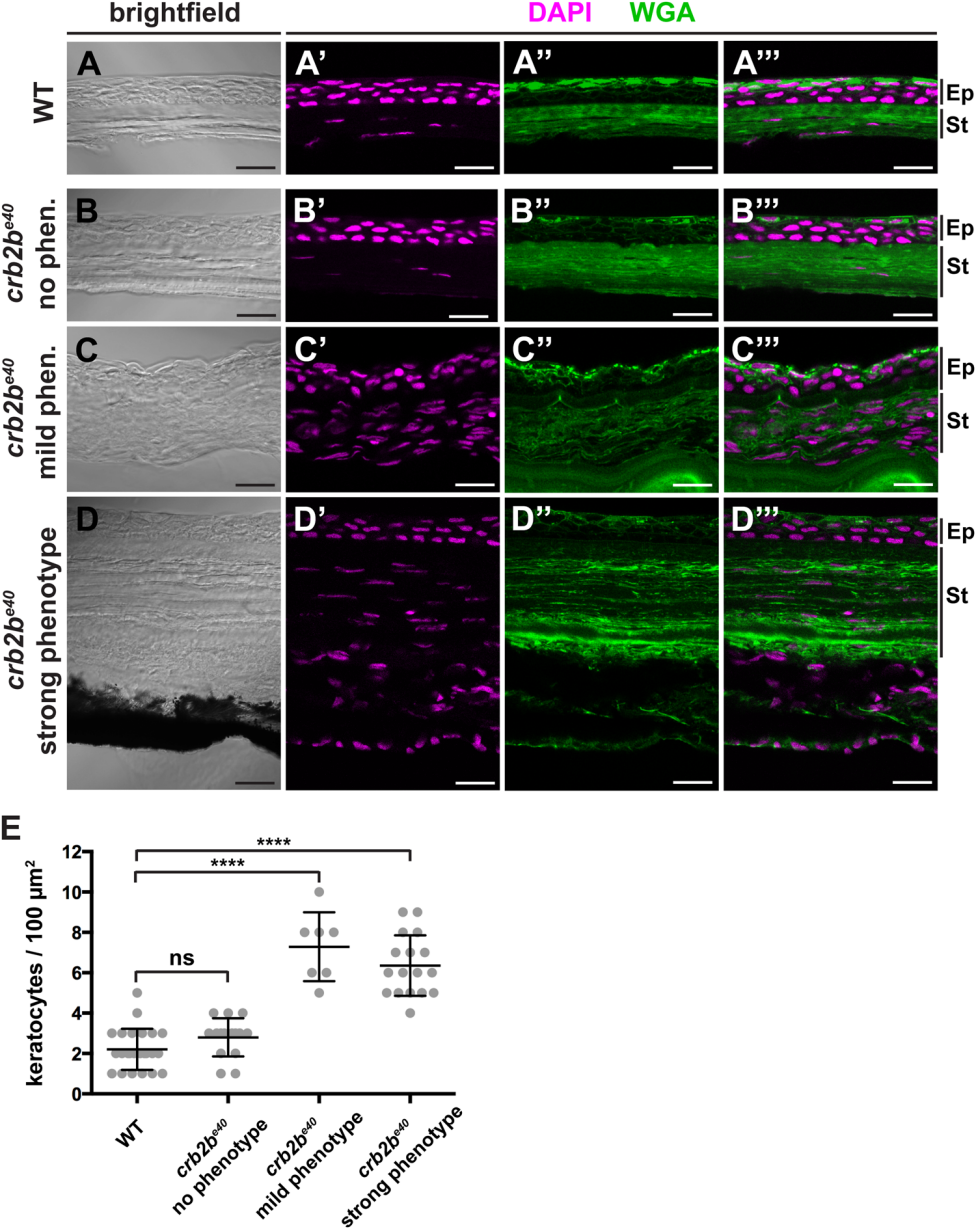
**Loss of Crb2b-lf leads to an increase in the number of stromal keratocytes in old fish.** (A–D‴). Brightfield (A,B,C,D), DAPI (magenta) and WGA (green) staining (A′–A″′,B′–B″′,C′-C″′,D′–D″′) of transverse cryosections of WT (A–A″′) and *crb2b^e40^* mutant (B–D″′) adult cornea. The stroma of *crb2b^e40^* mutants, which show an aberrant phenotype (C–D″′), appears to have more keratocyte nuclei (magenta) than mutants with no phenotype (B–B″′) or WT (A–A″′). Keratocyte nuclei were identified based on their flat and elongated morphology. Ep, corneal epidermis; St, corneal stroma. Scale bars: 5 µm. (E) Quantification of keratocyte nuclei in stromal tissue in WT versus *crb2b^e40^* mutant fish. The phenotypic categories correspond to the ones in A–D″′. Nuclei were counted from two separate 100 µm^2^ areas per adult fish in the central cornea, except for the ‘mild phenotype’ category due to lack of iris-free area at the centre of the cornea. Statistical significance was calculated by *t*-test (unpaired, with equal s.d., two-tailed). ****P<0.001; ns, not significant (*P=*0.0779).

Besides a defective corneal endothelium, TEM analysis suggested an excess of nuclei in the corneal stroma in *crb2b^e40^* eyes. Therefore, we quantified the number of stromal keratocytes by nuclear staining (DAPI, Fig. 7). Counterstaining with fluorescently-coupled wheat germ agglutinin (WGA) visualised the ECM within the cornea, helping to discriminate the different corneal layers. Keratocyte nuclei were counted from a central region of the cornea in WT (Fig. 7A–A″′) and mutant fish, which displayed either no obvious phenotype (Fig. 7B–B″′), a mild phenotype (iris does not reach the central cornea) (Fig. 7C–C″′) or a strong phenotype (pupil nearly closed) (Fig. 7D–D″′). In *crb2b^e40^* mutant fish that exhibited either a mild or a strong phenotype, the number of stromal keratocytes was significantly increased compared to that of WT fish (Fig. 7E). However, in eyes of *crb2b^e40^* fish that did not show an obvious mutant phenotype, the number of stromal keratocytes was normal (Fig. 7E). Together, these data show that loss of Crb2b-lf leads to an increased number of stromal keratocytes as well as defects in the corneal endothelium, while the ultrastructure of the corneal epithelium and fibre organisation within the stroma appear not to be affected.

### Loss of Crb2b-lf leads to lens capsule defects in old zebrafish

**Fig. 8.**
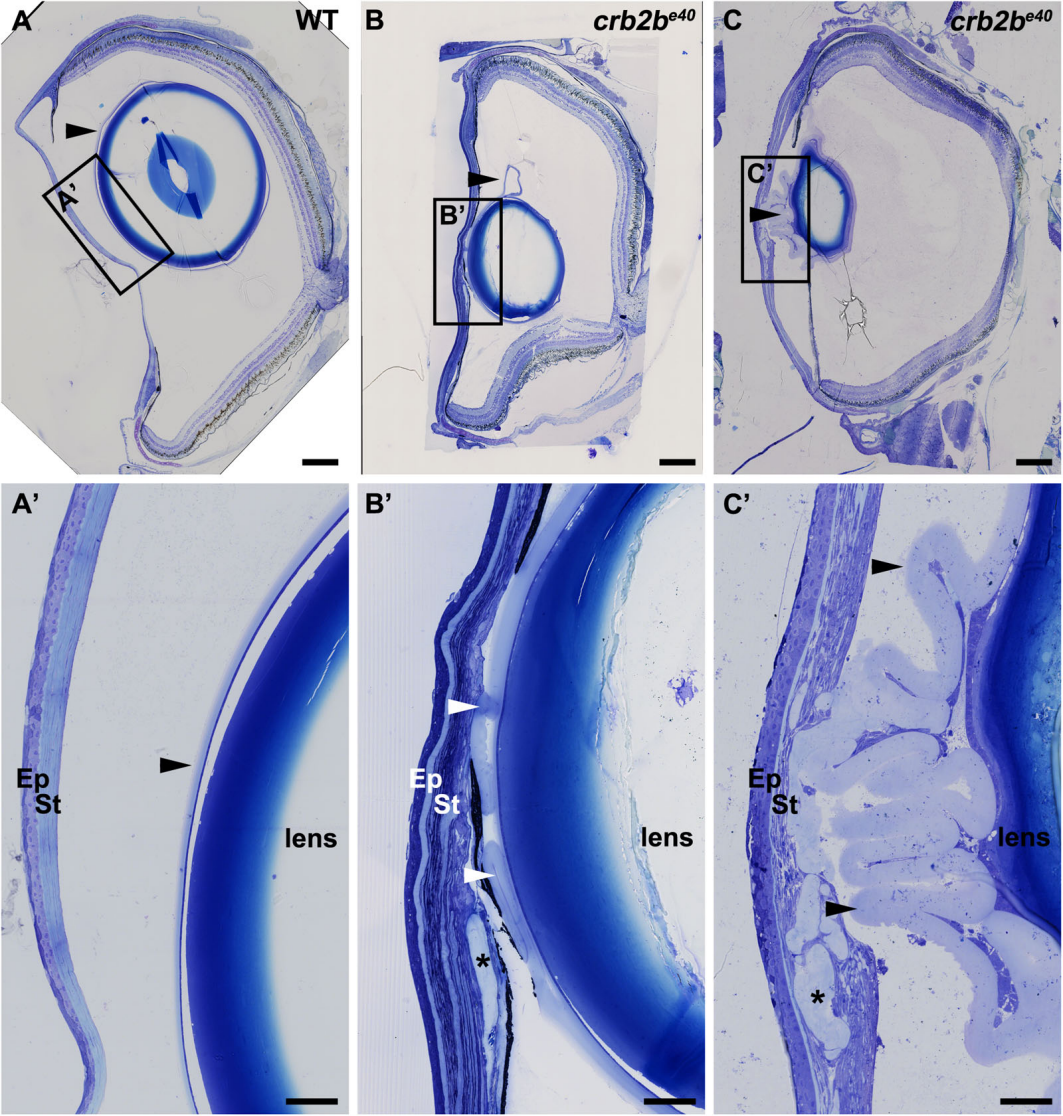
**Loss of Crb2b-lf leads to lens capsule abnormalities in old fish.** Toluidine Blue staining of transverse sections of WT (A,A′) and two *crb2b^e40^* mutant (B–C′) adult zebrafish eyes. In WT, the lens capsule is thin and smooth (A,A′, arrowheads) and is close to the lens. In mutant eyes (B–C′) the lens capsule appears thickened and folded (arrowheads). Asterisks mark areas where the lens capsule has penetrated into the cornea (B′,C′). Scale bars: (A–C) 200 µm; (A′–C′) 50 µm.

When dissecting *crb2b^e40^* mutant eyes, we discovered a complete loss of the lens in some eyes. In addition, analysis of eye histological sections revealed structural abnormalities in mutant lens capsules. In WT fish, the lens capsule, a thickened basement membrane deposited by the lens cells (Danysh and Duncan, 2009), forms a smooth layer completely surrounding the lens (Fig. 8A,A′, arrowheads). In most mutant fish, however, the lens capsule was expanded and highly folded (Fig. 8B–C′, arrowheads), occasionally penetrating into the layers of the corneal stroma (Fig. 8B′,C′, asterisks), while the lens epithelium appeared normal when compared to that of WT fish (data not shown).

Altogether, the data presented here indicate that Crb2b-lf plays a role in the maintenance of the AS. Its loss leads to overgrowth of the iris, increase in the number of the stromal keratocytes of the cornea, abnormalities in the corneal endothelium, and expansion of the lens capsule in old fish. The overall structure of the neural retina, however, was not affected in old *crb2b^e40^* mutant fish (Fig. S5). The AS phenotype appears to be age-dependent, as in young (5-month-old) *crb2b^e40^* adult fish the structures of the AS are normal (Fig. S6).

## DISCUSSION

Impaired vision due to abnormalities in either the neural retina (e.g. retinal degenerative diseases such as retinitis pigmentosa or LCA) or in the ocular AS (e.g. glaucoma, cataracts or AS dysgenesis) affects millions of people. Thus, understanding the way these structures develop and are maintained is of high priority. Crb proteins have been shown to have a conserved function in the retina, as in human, mouse and *Drosophila*, loss of Crb leads to degeneration of the PRCs and ultimately blindness. Here, we studied the function of one of the five *crb* orthologues, *crb2b*, in the zebrafish eye, by making use of a novel allele identified by TILLING (*crb2b^e40^*), which eliminates the long isoform of *crb2b*, Crb2b-lf. A comparable isoform has not been discovered for mammalian orthologues so far. For human CRB2, an alternative isoform lacking the transmembrane (TM) and the intracellular domains (ICD) has been described (Katoh and Katoh, 2004). For CRB1, three isoforms have been reported in the literature, one full-length isoform, one which lacks the TM and the ICD, and one which has a shortened ECD (deletion of three EGF-domains) (den Hollander et al., 2001, 1999; Pellissier et al., 2014).

Here, we found that old homozygous *crb2b^e40^* mutant fish exhibit several defects in the AS, including overgrowth of the tissues of the iris and the ICA, resulting in a decreased pupil size, and abnormalities in the lens and the cornea. Normal formation of apico-basal polarity and of the IS and OS of *crb2b^e40^* mutant PRCs was surprising, given previous results showing that MO-induced KD of *crb2b* leads to shortened PRC IS (Omori and Malicki, 2006). However, there is now ample evidence to conclude that genomic mutations can activate compensatory mechanisms during development, thus masking mutant phenotypes, which are otherwise induced by MO-mediated KD of the same gene (El-Brolosy et al., 2019; Rossi et al., 2015). The MOs used in the previous study are supposed to block the translation of both Crb2b-lf and Crb2b-sf, whereas the *crb2b^e40^* mutant still has an intact *crb2b-sf* sequence. However, a compensation by Crb2b-sf in the *crb2b^e40^* background during early development is unlikely, since, according to our data, expression of *crb2b-sf* is negligible during larval stages. This is in accordance with results from Zou et al., who also were not able to detect the *crb2b-sf* transcript in the developing retina using *in situ* hybridization (Zou et al., 2012). Since the paralog Crb2a is also expressed in the developing retina (Omori and Malicki, 2006), it could potentially compensate for the loss of Crb2b. However, we did not find any significant upregulation of the *crb2a* mRNA level in *crb2b^e40^* eyes or any obvious upregulation of Crb2a protein in larval retinas by immunofluorescence (data not shown). Furthermore, despite a normal morphology of *crb2b^e40^* mutant cones, we cannot exclude a functional defect in these cells. Additional studies testing the visual ability [e.g. the optokinetic response (Huang and Neuhauss, 2008; Zou et al., 2010) or the visual motor response (Emran et al., 2008)] of the mutant larva would be required to confirm normal PRC function.

Although zebrafish Crb2b proteins are expressed in adult PRCs, and human *CRB1* and *CRB2* and *Drosophila crb* protect against retinal degeneration (reviewed in Pichaud, 2018; Slavotinek, 2016), we did not observe any gross defects in the structure of the retina nor in the ultrastructure of PRCs in old *crb2b^e40^* fish. However, the retina of old *crb2b^e40^* mutant animals appeared somewhat thinner (data not shown), but the number of mutant fish was too low to quantify this phenotype. Based on data obtained upon expression of a dominant negative form of Crb2b, Zou et al. (2012) suggested that Crb2b mediates adhesion between the pentameric cone units in the adult retina, and long-term expression of this construct led to changes in planar patterning of cones as well as loss of cone PRCs (Fu et al., 2018). However, our preliminary analysis of the cone mosaic did not reveal any obvious defects in mutant fish. One possible explanation for the lack of a pronounced phenotype in adult *crb2b^e40^* retinas is a compensator role of Crb2b-sf in the absence of Crb2b-lf, but no increase in *crb2b-sf* mRNA level in the adult eye of *crb2b^e40^* mutants was observed. A further difference to the transgenic model is that the expression of dominant negative Crb2b-sf could also interfere with the function of Crb2a, which should not be affected in *crb2b^e40^* mutant fish.

Strikingly, we found that old *crb2b^e40^* fish reveal drastic abnormalities in the structures of the AS. The most obvious phenotype observed in eyes of old mutant fish was an expanded iris. During ocular development, the iris is generated from the tissues at the ICA (Soules and Link, 2005). Interestingly, we could show that Crb2b protein is expressed in the developing ICA tissue at 5 dpf. This is in accordance with the presence of *crb2b* mRNA in the developing ICA tissue at 7 dpf (Hsu et al., 2006). Our results suggest that these Crb2b-expressing cells are polarised, as they also express aPKC asymmetrically. Their apical compartments appear to abut each other in a sheet or rosette-like manner. Currently, the fate of these cells is unknown. The epithelial cells of the iris (pigmented and non-pigmented) and of the ciliary zone develop from the anterior margin of the retinal neuroepithelium, whereas iris stroma and the annular ligament are derived from the periocular mesenchyme (Soules and Link, 2005). The location of the Crb2b-expressing cells anterior to the RPE suggests that they could become part of the iris stroma or the annular ligament. On the other hand, their polarity rather indicates that they are epithelial in nature, and thus could be part of the developing iris epithelium, but without lineage-specific markers we cannot identify the nature of these cells conclusively. A zebrafish reporter line expressing a fluorescent protein under the *crb2b*-promoter would be a useful tool for tracking the lineage of these cells and their final fate and location, but this was outside the scope of this study.

Both the epi and the endothelium of the cornea are polarised tissues with apico-basal polarity and junctional complexes. It has previously been shown that zebrafish mutant for the polarity proteins Crb2a (*oko meduzy*, *ome*), aPKC (*heart and soul*, *has*), Mpp5 (*nagie oko*, *nok*) or Epb41l5 (*mosaic eyes*, *moe*) display abnormalities in the corneal epithelium and stroma at 3 dpf (Beyer et al., 2010). In these mutants, the epithelial cells do not properly adhere to each other, fluid-filled spaces are visible between the cells and the layered structure of the stroma is poorly formed. Furthermore, *CRB1* is expressed in the human corneal epithelium and patients with *CRB1* mutations exhibit corneal shape deviations (Beyer et al., 2010). The *CRB1* mutations in LCA patients have also been suggested to sensitise patients to keratoconus, a form of corneal dystrophy in which the cornea undergoes progressive thinning (McMahon et al., 2009). While we did not detect any abnormalities in the corneal epithelium of developing or adult *crb2b^e40^* mutant fish, the corneal endothelium, a normally monolayered tissue of flattened cells, appears multi-layered in the mutant. Due to the lack of cell type-specific markers, we were unable to determine the nature of these additional cells.

Endothelial defects in old *crb2b^e40^* mutant fish are associated with an increased number of keratocytes in the corneal stroma, while the orthogonal arrangement of collagen fibres is not affected. The normal appearance of collagen fibres in the mutant points to properly differentiated keratocytes, since the embryonic stroma only contains few collagen fibres (reviewed in Hassell and Birk, 2010). It is known that the endothelium regulates the transparency and the lamellar organisation of the stroma (Beyer et al., 2010). Whether the mis-organised endothelium in *crb2b^e40^* mutant fish is causing the defects in the stroma, or whether the mutant phenotypes in these two tissues develop in parallel due the common origin of endothelial and stromal cells from the periocular mesenchyme (Soules and Link, 2005), remains to be explored. Alternatively, the increase in stromal keratocytes could be due to a defect in the limbal stem cell niche, which is the source of corneal and stromal cells in mature animals (Funderburgh et al., 2016; Gonzalez et al., 2018; Yazdanpanah et al., 2017). In zebrafish, the functionality of the limbus has been demonstrated by lineage-tracing of post-developmental corneal cells (Pan et al., 2013; Soules and Link, 2005; Zhao et al., 2006), but the limbal stem cell niche is yet to be characterized. Due to the lack of appropriate cell-type specific markers the expression of Crb2b in the limbus can currently not be addressed. Further experiments will elucidate whether the abnormalities observed in the mutant stroma impact on the transparency of the cornea, which acts as a refractive lens and hence is instrumental for proper vision.

Finally, old *crb2b^e40^* fish exhibited abnormalities in the lens, ranging from capsule defects to complete loss of the lens in some fish. In mutant eyes, the lens capsule, a basement membrane that completely covers the lens, appeared expanded and occasionally penetrated into the corneal tissues. The lens capsule is deposited on the basal side of the lens epithelium, which in many cases appears normal in *crb2b^e40^* fish. The lens capsule protects the lens from infection, functions as a reservoir for lens growth factors and participates in the exchange of metabolites between the avascular lens and its environment. Hence, defects in its organisation could potentially influence the growth of other tissues of the AS (reviewed in Danysh and Duncan, 2009). In other zebrafish mutants, in which the structure of the lens capsule is disturbed, such as *laminin alpha 1* (*lama1*) (Pathania et al., 2014), *laminin beta 1* (*lamb1*) and *laminin gamma 1* (*lamc1*) (Lee and Gross, 2007) or *occhiolino* (*occ*) mutants (Aose et al., 2017), the lens develops abnormally or is completely lost. In addition, in the *occ* (Aose et al., 2017), *arl* (*arrested lens*) and *dsl* (*disrupted lens*) mutants aberrant lens development and lens degeneration is accompanied by a small pupil phenotype (Vihtelic et al., 2001). Thus, a lens abnormality can compromise the integrity of the anterior chamber, which in turn influences the other AS tissues. The expanded lens capsule observed in *crb2b^e40^* mutant fish bears some similarity to a phenotype found in patients with true exfoliation syndrome, an age-related disease often linked to glaucoma, in which the anterior lens capsule delaminates from the lens (Teekhasaenee, 2018; Zhou et al., 2012). A more detailed analysis of the lens phenotype and its development in *crb2b^e40^* mutant fish will confirm possible further similarity to human disease.

Overall, the *crb2b^e40^* mutant phenotype is complex and influences the growth of multiple AS structures, which originate from different cell lineages. Whether the abnormalities observed in the different parts of the AS develop independently from each other, or whether defects in one tissue affect the others, has to be determined. Tissue-specific knockdown of Crb2b in the individual structures of the AS would help in understanding the origin of the phenotype, but is currently not feasible due to the sparse knowledge of the molecular biology of the AS tissues.

Unfortunately, we were unable to detect Crb2b protein expression in the adult AS with either of our Crb2b antibodies, possibly due to a low expression level, since Crb2b expression could be visualised in PRCs. Another technical barrier is the presence of pigment cells in the iris, which mask potential antibody signals within the iris stroma and the ciliary zone, even in an albino background. Generating a *crb2b*-reporter line could help to solve this problem. However, data from gene expression analysis published previously show that *crb2b* is expressed in adult AS structures (cornea and/or iris) (Takamiya et al., 2015). Given the conservation of Crb proteins in vertebrates, including human, the novel role of zebrafish *crb2b* described here may stimulate further studies on the function of polarity regulators in ocular diseases originating in AS tissues.

## MATERIALS AND METHODS

### Zebrafish strains, transgenic lines and husbandry

Adult zebrafish (*Danio rerio*) were maintained at 26.5°C under standard conditions in a 14 h/10 h light/dark cycle. All embryos were raised in E3 medium in a dark incubator at 28.5°C until 5 dpf. Staging of fish was done according to Kimmel et al. (1995) and Parichy et al. (2009). For the experiments in WT fish, the AB strain was used. The Tg(*bactin*:mRas-EGFP) [current ZFIN name Tg(*Ola.Actb*:*Hsa*.*HRAS-EGFP*)] (Cooper et al., 2005) line has been described before. All animal studies were performed in accordance with European and German animal welfare legislation. Protocols were approved by the Institutional Animal Welfare Officer, and necessary licenses were obtained from the regional Ethical Commission for Animal Experimentation of Dresden, Germany (Tierversuchskommission, Landesdirektion Sachsen).

### Generation and genotyping of the *crb2b^e40^* mutant line

To isolate point mutations of the zebrafish *crb2b* locus, we applied the TILLING approach and screened a library of 3840 zebrafish that carry random point mutations induced by ENU treatment (de Bruijn et al., 2009; Kettleborough et al., 2011; Winkler et al., 2011). Oligonucleotides against exon 2 of *crb2b* were designed to identify an early nonsense mutation of this gene (forward outer primer: TCTTAATCAGAGTATTGTCTTAATCG, reverse outer primer: TCTCATGCTAGATGTCAGTGTG, forward inner primer: TGTAAAACGACGGCCAGTACGTAGCCGTTGATCAATAA, reverse inner primer: AGGAAACAGCTATGACCATCAGCTTTGAAGAAGCTAATTT). Exon 2 was amplified from genomic zebrafish DNA in a nested PCR approach, and Sanger sequencing of PCR fragments was performed using the universal sequencing primer AGGAAACAGCTATGACCAT. All point mutations were identified by the PolyPhred software tool (Stephens et al., 2006). Primary hits, leading to potentially deleterious mutations, were re-sequenced in an independent approach and verified. Among them, one nonsense mutation in exon 2 (nt 349 on NM_001045162.1, nt 2857915 on NC_007119.7) of zebrafish *crb2b* (*crb2b^e40^*) was verified and the founder fish was isolated from the living zebrafish library. Heterozygous *crb2b^e40^* zebrafish were further outcrossed to WT for several generations and then bred to homozygosity. Molecular genotyping of *crb2b^e40^* founders was done applying the TILLING screening protocol.

### Generation of rabbit anti-Crb2b antibody

For the rabbit anti-Crb2b^e8e9^ polyclonal antibody, animals were co-immunized with two Crb2b peptides. Peptide 1 contained Crb2b (NP_001038627.1) aa 564-579 (CTRGGYLLIDLHHKNNR, exon 8) and peptide 2, aa 810-823 (CRHLNGLVLQLQHDN, exon 9). The rat anti-Crb2b^lf^ antibody, which recognises an epitope in the N-terminus of Crb2b-lf (aa 1-71, AFK80351.1; for location of the epitopes, see Fig. 1A), has been described before (Crespo and Knust, 2018). Peptides were produced by Peptide Specialty Laboratories GmbH, Heidelberg, and immunisations were done by Charles River Laboratories, Germany.

### Sample preparation for cryosectioning

For immunostaining, embryos were treated with phenyl thiourea (PTU, 0.2 mM) to prevent pigmentation from 22 hpf on. Larvae were sacrificed with an overdose of MESAB (ethyl 3-aminobenzoate methanesulfonate, Sigma-Aldrich), and fixed with ice-cold 4% PFA in PBS overnight at 4°C. The next day, samples were washed with PBS with 0.1% Tween-20 (PBST) and processed through 10%, 20% and 30% sucrose solutions. Samples were incubated in 1:1 30% sucrose, 50% NEG-50 (Thermo Fisher Scientific) and then embedded in NEG-50 and stored at −80°C. For adult eyes, zebrafish were sacrificed with an overdose of MESAB and decapitated. Zebrafish eyes were dissected and fixed in 4% PFA overnight at 4°C. After fixation, samples were washed 2×5 min in PBS and kept for 1 h in 5% sucrose in 1×PBS, and then incubated overnight at 4°C in 30% sucrose in 1×PBS, followed by a second overnight incubation at 4°C in 1:1 solution of 30% sucrose in 1×PBS/NEG-50™ (Thermo Fisher Scientific). Finally, the samples were incubated for 1 h at room temperature (RT) in NEG-50™, mounted and frozen in dry ice. All samples were kept at −80°C until sectioning. Cryosections were generated using a Microm HM 560 (Thermo Fisher Scientific) at 16–20 µm, and left to dry for at least 2 h at room temperature. After sectioning, all samples were kept at −20°C until use.

### Immunostaining of cryosections

Cryosections were thawed at RT for 2 h prior to staining. Slides were washed in PBS for 2×10 min at room temperature. For aPKC and ZO-1 stainings, antigen retrieval was done by incubating sections in 10 mM sodium citrate, 0.05% Tween-20 (pH 6) for 20 min at 70°C. Slides were allowed to cool to RT in antigen retrieval buffer for a further 30 min and were then washed 3×5 min with PBST. Samples were permeabilized with 0.1% SDS in PBS for 15 min, washed 3×5 min with PBST, blocked in 10% normal horse serum (NHS) in 0.3% Triton X-100 in PBS for a minimum of 1 h, and then stained overnight with the primary antibody in 1% BSA, 1% NHS, 0.3% Triton X-100 in PBS at 4°C. On the second day, slides were washed 3×20 min in PBST (0.1% Triton X-100 in PBS) and incubated overnight with the secondary antibody in 1% bovine serum albumin (BSA), 1% NHS, 0.3% Triton X-100 in PBS at 4°C. The following primary antibodies were used: mouse monoclonal anti-Zpr1 (1:200, ZIRC), rabbit polyclonal anti-aPKC (1:50, C-20, sc-216-G, Santa Cruz Biotechnology), mouse monoclonal anti-ZO-1 (1:200, #339100, Molecular Probes), mouse monoclonal anti-Crb2a (1:50, zs-4, ZIRC), rabbit polyclonal anti-Crb2b^e8e9^ (1:50, this study), rat polyclonal anti-Crb2b^lf^ [1:50, (Crespo and Knust, 2018)], mouse monoclonal anti-acetylated tubulin (1:250, T6793, Sigma-Aldrich). Secondary antibodies (Alexa Fluor 488 or 568 conjugates, Invitrogen) were used at 1:500. Alexa Fluor 488 phalloidin (1:100, Invitrogen), Alexa Fluor 660 phalloidin (1:20, Invitrogen), Alexa Fluor 488 conjugated WGA (1:200, Molecular Probes) and DAPI (Roche) were added together with the secondary antibodies. After secondary antibody staining, samples were washed 2×20 min in PBST, 2×20 min in PBS and finally mounted in Vectashield antifade mounting medium (Vector Laboratories). Slides were kept at 4°C until imaging. A minimum of three individuals per sample group were analysed for each staining.

### Histology by light and TEM

For semi and ultrathin plastic sections, zebrafish larvae were anesthetised in MESAB and heads were dissected in ice-cold PBS. Larval heads were fixed with 2% PFA, 2% glutaraldehyde in 50 mM Hepes buffer (pH 7.25) for 1 h at RT and further overnight at 4°C. Adult fish were sacrificed with an overdose of MESAB and decapitated, after which heads were cut in half across the midline and unnecessary tissue was dissected away to provide a better access of fixative to the ocular tissues. Samples were fixed with 2% PFA, 2% glutaraldehyde in 50 mM Hepes buffer (pH 7.25). Fixation of half heads was enhanced in the beginning of fixation by a microwave treatment with vacuum cycle (Pelco Biowave Pro, Ted Pella, Inc.): 100 W, 2 min, 25°C; cooling 2 min on ice; 100 W, 2 min, 25°C. Samples were then fixed further overnight at 4°C. The next day, samples were washed in 100 mM Hepes buffer (5×10 min) and then in 2×5 min in PBS. 1% OsO_4_, 1.5% potassium ferrocyanide in PBS was added to the samples and incubated at RT for 90 min. All samples were washed first in PBS (3×5 min), then in distilled water (4×5 min) and dehydrated in a series of EtOH (30%, 50%, 70%, 90% and 100%, for 20 min in each solution). Sections were then incubated at RT in 100% propidium oxide (Sigma-Aldrich) for 2×10 min, 2:1 propidium oxide:Durcupan (Sigma-Aldrich) for 45 min and 1:1 propidium oxide:Durcupan for 1.5 h. Larval heads were incubated in 1:2 propidium oxide:Durcupan for 2 h and in 100% Durcupan overnight. The next day, Durcupan was exchanged and samples were incubated for an additional 4–5 h before mounting in 100% Durcupan. Adult half heads were incubated in 1:2 propidium oxide:Durcupan overnight at 4°C, followed by 100% Durcupan for 4–5 h at RT. Then Durcupan was exchanged and samples incubated in 100% Durcupan overnight at 4°C. The next day, Durcupan was again exchanged and samples were incubated for further 4–5 h before mounting in 100% Durcupan. For TEM, 70 nm sections were stained with 2% uranyl acetate (Polysciences, Inc) in water for 10 min, followed by 5 min in lead(II) citrate tribasic trihydrate (Sigma-Aldrich). For light microscopy, 500 nm or 1 µm semithin plastic sections were stained with 0.05% Toluidine Blue, 1% sodium borate solution in a water bath at 40°C, rinsed with distilled water, destained with 70% ethanol, rinsed with distilled water and finally mounted in Entellan (Merck). A minimum of three individuals per sample group were analysed. TEM images were obtained with a Morgani 268 TEM (FEI), using a Morada CCD Camera (SIS/Olympus).

### Image acquisition and analysis

All samples immunostained were imaged using either a Zeiss multiphoton laser scanning upright microscope (Axio examiner.Z1) with Zeiss Plan-Neofluar 40×NA 0.8 or Zeiss Plan-Neofluar 63×NA 0.8 objectives, or with a Zeiss LSM 880 confocal microscope with 63×Zeiss LCI Plan-Neofluar 1.3 objective. All confocal images were acquired using ZEN 2011 Black software from Zeiss. Toluidine Blue stainings were imaged using a Zeiss ApoTome ImagerZ.1 microscope using ZEN Blue software (Zeiss). For TEM, samples were imaged with a Morgagni TEM (80 kV) using a Morada CCD camera (EMSIS GmbH) and ITEM software (EMSIS GmbH). Brightfield images of larval and adult zebrafish were imaged using an Olympus SZ61 microscope and an Olympus U-TV0.5XC-3 camera. Images were processed using Fiji (Schindelin et al., 2012) and Photoshop (Adobe). Graphs were plotted and the statistical analysis was done with GraphPad Prism 6 (GraphPad Software).

### RNA extraction, cDNA synthesis and qPCR

Zebrafish were sacrificed with an overdose of MESAB, eyes dissected in ice-cold PBS and homogenised in Trizol (Ambion), first using a pestle and then a syringe and a needle. Total RNA was extracted according to the manufacturer's protocol. 0.5–1 µg of total RNA was treated with DNase I (NEB) and transcribed to cDNA using oligo(dT)_12-18_ (Invitrogen) and random hexamer (Invitrogen) primers with the SuperScript III reverse transcriptase (Invitrogen) according to the manufacturer's protocol. Primers used for mRNA quantification had the following sequences: *crb2b-lf*, F 5′-CCATGGAGGAGTCTGTCTGG-3′, R 5′-GGGTCAGAAGAACACTCGGG-3′; *crb2b-sf*, F 5′-GTTTGGCAGGATGAGAGGACT-3′, R 5′-CGTTCTGACACGGATCACTCT-3′; and *rpl13a*, F 5′-TCTGGAGGACTGTAAGAGGTATGC-3′, R 5′-CTGCTTGTGACTTGTGTGTTCC-3′ (Tang et al., 2007). qPCR reactions were set up using the ABsolute SYBR Green QPCR Mix (Thermo Fisher Scientific) and run with the Mx3000P cycler (Stratagene). Three or four biological replicates were analysed for each sample group. Delta_Ct_ values were calculated by C_t(*GOI)*_–C_t(*rpl13a*)_, where GOI stands for gene of interest. Graphs depicting the data were drawn and statistical analysis performed with GraphPad Prism 6 (GraphPad Software).

## Supplementary Material

Supplementary information
