# Supplementary material for: Loss of Crb2b-lf leads to anterior segment defects in old zebrafish
Source: Biol Open. 2020 Feb 11;9(2):bio047555. doi: 10.1242/bio.047555 (PMC7044448; doi:10.1242/bio.047555)
Supplement: Supplementary information [file biolopen-9-047555-s1.pdf]

Figure S1.

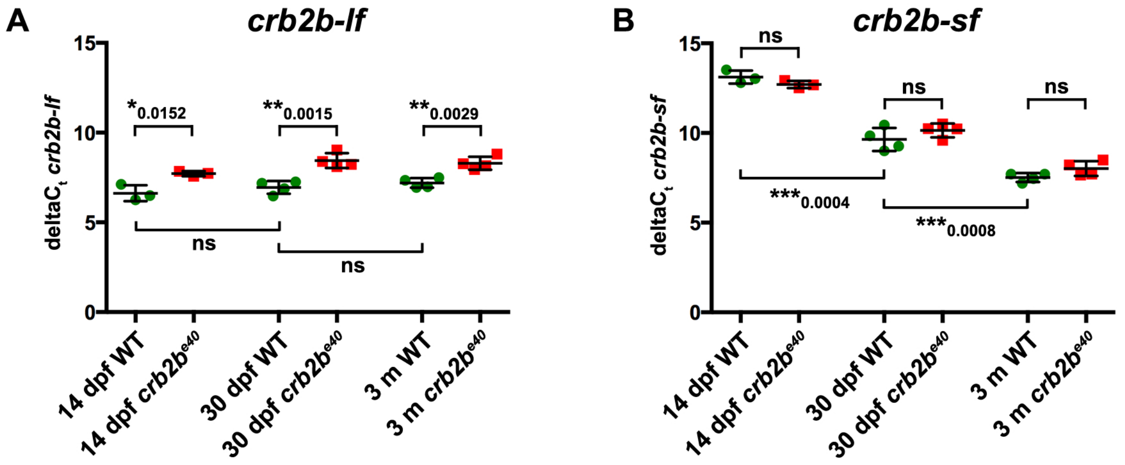

Supplemental Figure S1. *crb2b-sf* mRNA expression is upregulated after larval stages. A. *crb2b-lf* expression in eye extracts from late developmental stages (14 dpf, 30 dpf) and from adults of 3 months (3 m) in WT (green) versus *crb2b*^{e40} mutant (red) fish. No significant change is detected in *crb2b-lf* levels during maturation. *crb2b*^{e40} mutants have consistently lower levels of *crb2b-lf* transcripts. **B.** *crb2b-sf* expression in eye extracts from late developmental stages (14 dpf, 30 dpf) and from adults (3 m) in WT versus *crb2b*^{e40} mutant fish. *crb2b-sf* transcript levels are upregulated during maturation. *crb2b-sf* levels are not changed in *crb2b*^{e40} mutants. Graphs show mean delta C_t values normalised to the housekeeping gene *rpl13a* +/- standard deviation. Statistical significance of difference in mean delta C_t values was calculated by *t*-test (unpaired, with equal s.d., two-tailed). A lower delta C_t value indicates upregulation of mRNA. Values next to asterisks denote p values. ns, non-significant.

Figure S2.

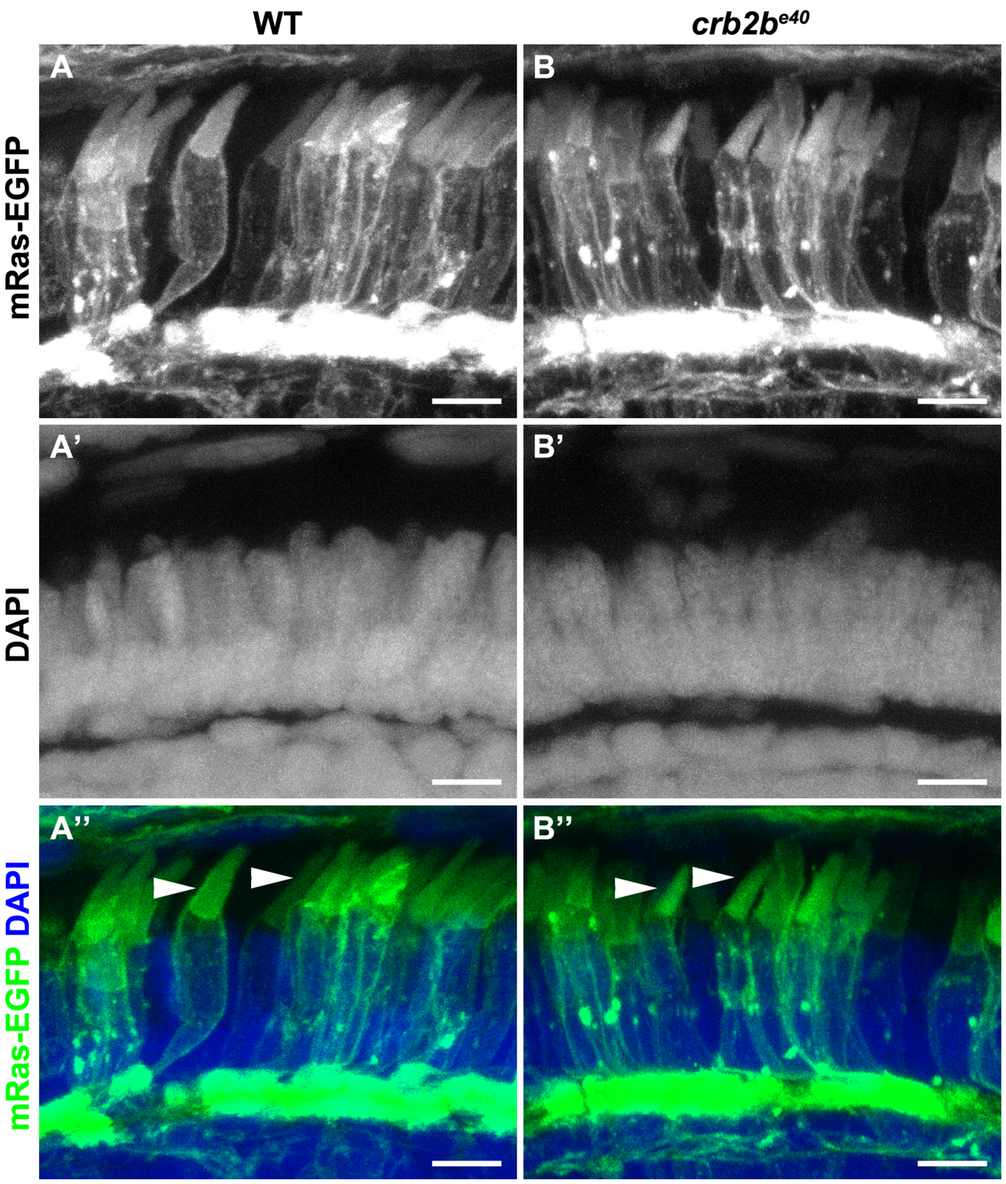

Supplemental Figure S2. Outer segments (OSs) extend normally in *crb2b^{e40}* mutant larvae. Transverse cryosections through WT (A-A'') and *crb2b^{e40}* mutant (B-B'') eyes in Tg(*bactin*:mRas-EGFP) background at 5 dpf. **A-A''.** Membrane-targeted EGFP visualizes the OS compartment (arrowheads in A'') in WT photoreceptor cells (PRCs). **B-B''.** *crb2b^{e40}* mutant PRC OSs (arrowheads in B'') do not appear different from WT OSs. Scale bars, 5 µm.

Figure S3.

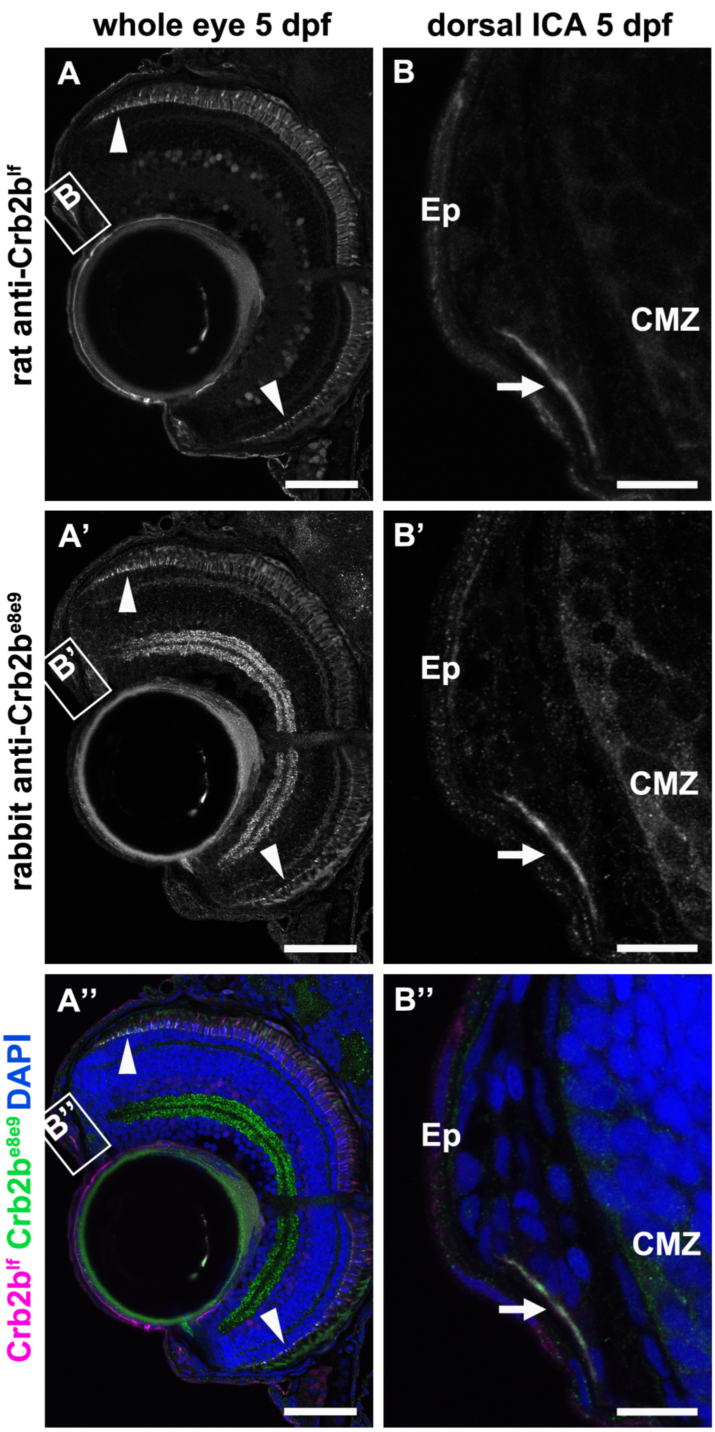

Supplemental Figure S3. Expression pattern of Crb2b in the anterior segment. Transverse cryosections through WT eyes at 5 dpf co-immunostained with rat anti-Crb2b^{lf} (A, A'', B, B'') and rabbit anti-Crb2b^{e8e9} (A', A'', B', B''). **A-A''.** Overview of the larval eye. Both antibodies stain an epitope in the PRC layer, which is especially visible in the newly formed PRCs (arrowheads). **B-B''.** In the dorsal iridocorneal angle (ICA) an additional domain of Crb2b expression is detected with both antibodies (arrows). CMZ, ciliary marginal zone; Ep, epidermis. Scale bars: A-A'', 50 µm; B-B'', 10 µm.

Biology Open • Supplementary information

Figure S4.

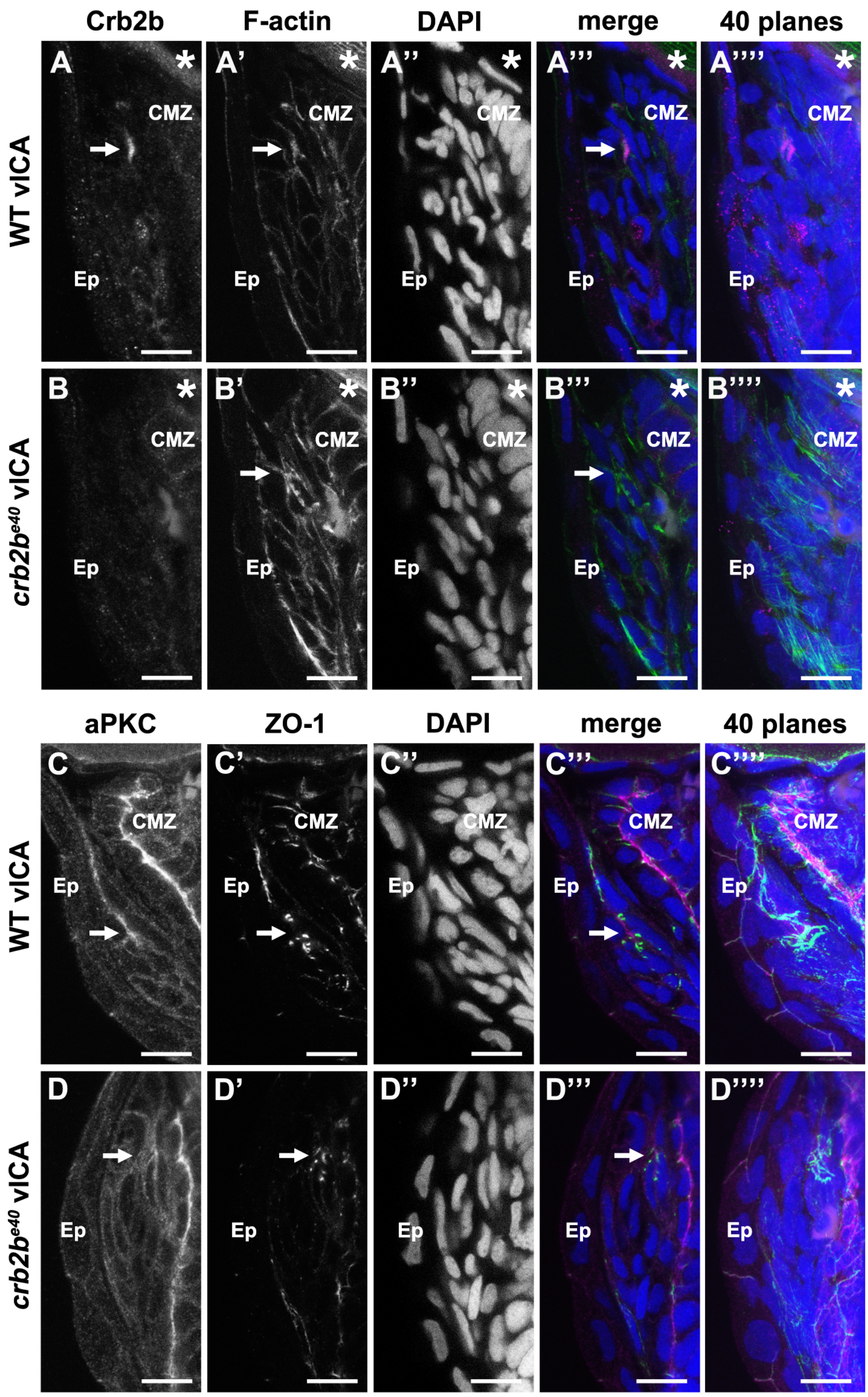

Supplemental Figure S4. Crb2b is expressed in polarised cells of the ventral iridocorneal angle at 5 dpf. Immunostaining of transverse cryosections, depicting the ventral iridocorneal angles (ICA) of a WT (A-A'''', C-C'''') and a *crb2b*^{e40} mutant eye (B-B''', D-D''''). Anti-Crb2b^{e8e9} staining (magenta in merged images) detects in a cluster of cells in the WT ICA (A) but not in the mutant ICA (B). Phalloidin visualises F-actin (green in merged images) that appears apically enriched (A', B'). aPKC (C, D; magenta in merged images) and ZO-1 (C', D'; green in merged images) are also localised apically in these cells. Arrows point to the cluster of polarised, Crb2b expressing cells, and asterisks mark the lens. vICA, ventral iridocorneal angle; Ep, epidermis; CMZ, ciliary marginal zone. Scale bars, 10 µm.

Biology Open • Supplementary information

Figure S5.

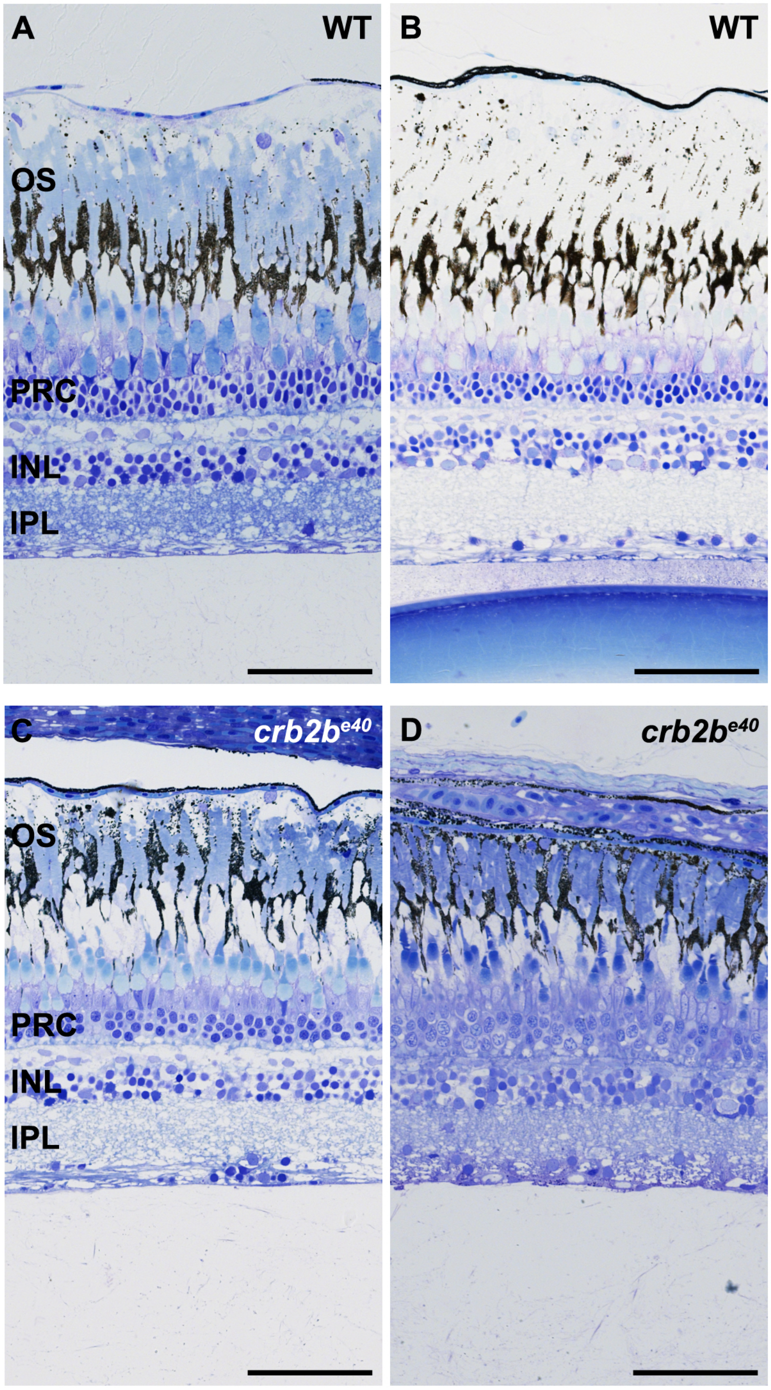

Supplemental Figure S5. Layering of the neural retina is normal in old *crb2b^{e40}* fish. Toluidine blue staining of transverse sections of old adult WT (A, B) and *crb2b^{e40}* mutant (C, D) fish. In the mutant (C, D) retinal layering appears normal in comparison to WT control retina (A, B). The figure shows two examples of different individuals for each WT and mutant. Mutant eyes had anterior segment defects. Retinal tissue was imaged on the dorsal side of the optic nerve. IPL, inner plexiform layer; INL, inner nuclear layer; PRC, photoreceptor cell bodies; OS, outer segments. Scale bars, 50 µm.

Biology Open • Supplementary information

Figure S6.

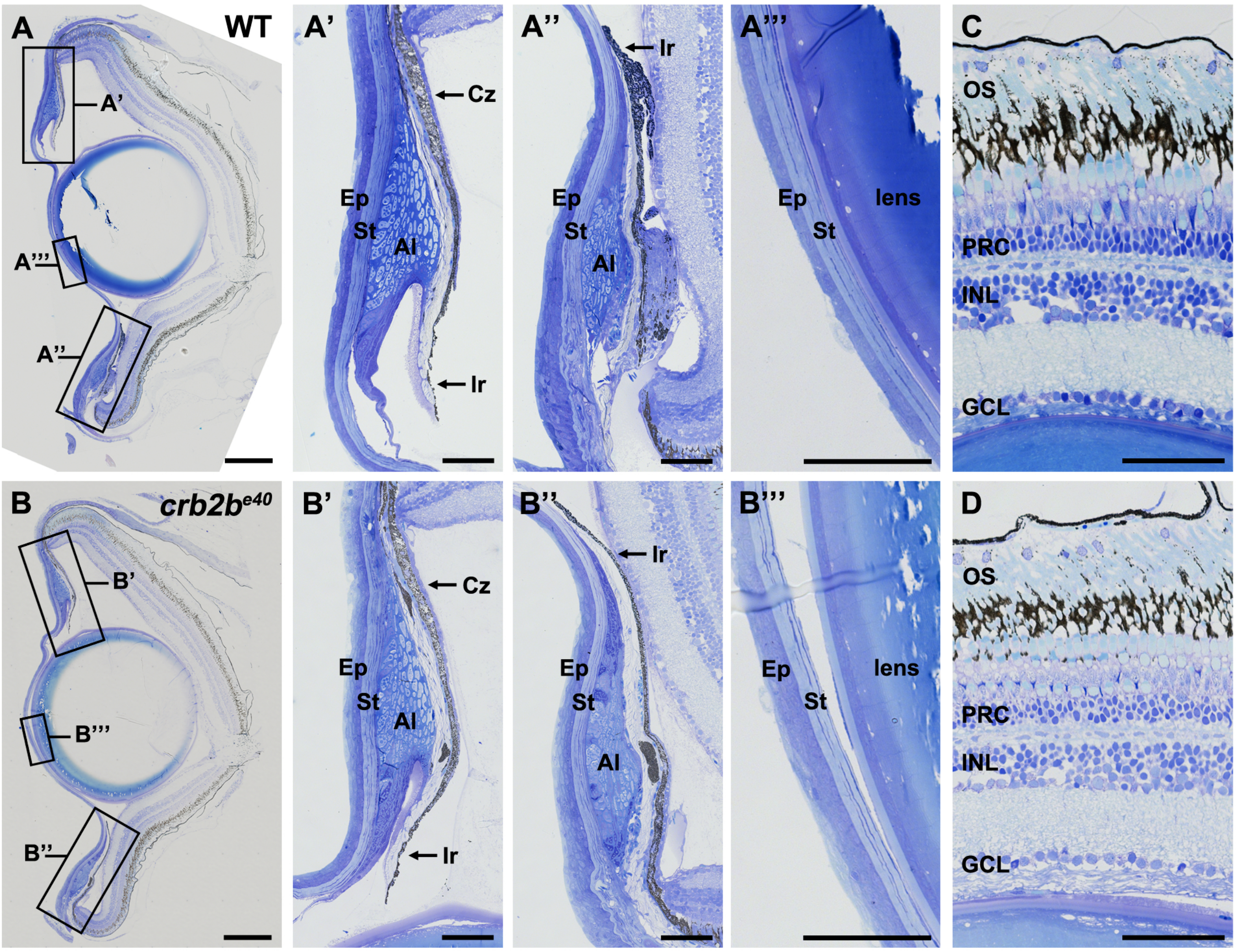

Supplemental Figure S6. The eyes of young *crb2b^{e40}* mutant adult fish appear normal. Toluidine blue stained transverse sections of WT (A-A''', C) and *crb2b^{e40}* (B-B''', D) eyes of 5-month-old fish. In mutant eyes, the dorsal (B') and ventral (B'') iridocorneal angle as well the cornea and the lens capsule (B''') appear normal (compare to A'-A'''). Cells of the mutant neural retina (D) do not display any apparent differences in comparison to WT cells (C). Retinal tissue was imaged dorsal to the optic nerve. Ep, corneal epithelium; St, corneal stroma; Al, annular ligament; Ir, iris; Cz, ciliary zone; GCL, ganglion cell layer; INL, inner nuclear layer; PRC, photoreceptor cell bodies; OS, outer segments. Scale bars: A, B, 200 µm; A'-A''', B'-D, 50 µm.

Biology Open • Supplementary information
